# Genomic and environmental risk factors for cardiometabolic diseases in Africa: methods used for Phase 1 of the AWI-Gen population cross-sectional study

**DOI:** 10.1080/16549716.2018.1507133

**Published:** 2018-09-27

**Authors:** Stuart A. Ali, Cassandra Soo, Godfred Agongo, Marianne Alberts, Lucas Amenga-Etego, Romuald P. Boua, Ananyo Choudhury, Nigel J. Crowther, Cornelius Depuur, F. Xavier Gómez-Olivé, Issa Guiraud, Tilahun N. Haregu, Scott Hazelhurst, Kathleen Kahn, Christopher Khayeka-Wandabwa, Catherine Kyobutungi, Zané Lombard, Felistas Mashinya, Lisa Micklesfield, Shukri F. Mohamed, Freedom Mukomana, Seydou Nakanabo-Diallo, Hamtandi M. Natama, Nicholas Ngomi, Engelbert A. Nonterah, Shane A. Norris, Abraham R. Oduro, Athanase M. Somé, Hermann Sorgho, Paulina Tindana, Halidou Tinto, Stephen Tollman, Rhian Twine, Alisha Wade, Osman Sankoh, Michèle Ramsay

**Affiliations:** a Sydney Brenner Institute for Molecular Bioscience, Faculty of Health Sciences, University of the Witwatersrand, Johannesburg, South Africa; b Faculty of Health Sciences University of the Witwatersrand, Division of Human Genetics, National Health Laboratory Service and School of Pathology, Johannesburg, South Africa; c Navrongo Health Research Centre, Navrongo, Ghana; d Department of Pathology and Medical Science, School of Health Care Sciences, Faculty of Health Sciences, University of Limpopo, Polokwane, South Africa; e Clinical Research Unit of Nanoro, Institut de Recherche en Sciences de la Sante, Nanoro, Burkina Faso; f Department of Chemical Pathology, National Health Laboratory Service, Faculty of Health Sciences, University of the Witwatersrand, Johannesburg, South Africa; g MRC/Wits Rural Public Health and Health Transitions Research Unit (Agincourt), School of Public Health, Faculty of Health Sciences, University of the Witwatersrand, Johannesburg, South Africa; h African Population and Health Research Center, Nairobi, Kenya; i School of Electrical & Information Engineering, University of the Witwatersrand, Johannesburg, South Africa; j School of Public Health, Faculty of Health Sciences, University of the Witwatersrand, Johannesburg, South Africa; k INDEPTH Network, Accra, Ghana; l MRC/Wits Developmental Pathways for Health Research Unit, Faculty of Health Sciences, University of the Witwatersrand, Johannesburg, South Africa; m Statistics Sierra Leone, Tower Hill, Freetown, Sierra Leone; n Department of Community Medicine, College of Medicine and Allied Health Sciences,University of Sierra Leone, Freetown, Sierra Leone

**Keywords:** Cardiometabolic disease, African populations, burden of disease, H3Africa, AWI-Gen

## Abstract

There is an alarming tide of cardiovascular and metabolic disease (CMD) sweeping across Africa. This may be a result of an increasingly urbanized lifestyle characterized by the growing consumption of processed and calorie-dense food, combined with physical inactivity and more sedentary behaviour. While the link between lifestyle and public health has been extensively studied in Caucasian and African American populations, few studies have been conducted in Africa. This paper describes the detailed methods for Phase 1 of the AWI-Gen study that were used to capture phenotype data and assess the associated risk factors and end points for CMD in persons over the age of 40 years in sub-Saharan Africa (SSA). We developed a population-based cross-sectional study of disease burden and phenotype in Africans, across six centres in SSA. These centres are in West Africa (Nanoro, Burkina Faso, and Navrongo, Ghana), in East Africa (Nairobi, Kenya) and in South Africa (Agincourt, Dikgale and Soweto). A total of 10,702 individuals between the ages of 40 and 60 years were recruited into the study across the six centres, plus an additional 1021 participants over the age of 60 years from the Agincourt centre. We collected socio-demographic, anthropometric, medical history, diet, physical activity, fat distribution and alcohol/tobacco consumption data from participants. Blood samples were collected for disease-related biomarker assays, and genomic DNA extraction for genome-wide association studies. Urine samples were collected to assess kidney function. The study provides base-line data for the development of a series of cohorts with a second wave of data collection in Phase 2 of the study. These data will provide valuable insights into the genetic and environmental influences on CMD on the African continent.

## Background

Sub-Saharan Africa (SSA) is in the throes of an epidemiological and health transition, described conceptually by the complex patterns of change in health and disease, and interactions with demographic, dietary, economic and social determinants and their consequences[1–3]. Many recent studies have focused largely on South Africa as a proxy for the region [4–9] but this approach can make it difficult to unpack precisely where countries within SSA lie on the spectrum of the transition. Recently, robust population-based data generated by INDEPTH (International Network for the Demographic Evaluation of Populations and Their Health in low- and middle-income countries) and its partners have provided insight into the extent to which SSA is experiencing an epidemiological change [10,11]. Examining 70,810 verbal autopsies (VA) from 80,880 deaths from 10 field centres across seven countries in the region, they provide some of the first empirical evidence from individual countries in SSA of increasing mortality because of non-communicable diseases (NCD). As an outcome measure for NCD, verbal autopsies have provided a powerful means to track whether countries within the region are achieving the goals set out by the World Health Organization (WHO) 25 × 25 targets. These include a 25% reduction in premature NCD mortality resulting from the four major NCDs (cardiovascular diseases, cancer, diabetes and chronic respiratory diseases) by 2025 [12], including a 25% relative reduction in prevalence of raised blood pressure, and aims to halt the rise in diabetes and obesity. However, complementary large-scale harmonized data on risk factor and morbidity burden, needed to address the latter target, are still lacking from Africa.

A number of studies aim to advance health in Africa through supporting research into healthy life trajectories. One study in South Africa focuses on reducing transgenerational risk of obesity and metabolic disease in the disadvantaged urban area of Soweto in Johannesburg, and is called ‘Building Knowledge and a Foundation for Healthy Life Trajectories in South Africa: A Preconception Developmental Origins of Health and Disease (DOHaD) Intervention Cohort’ [13,14]. The study represents a vulnerable population and is a community-based intervention study that aims to improve the health and well-being of disadvantaged women before and during pregnancy to improve health outcomes in their children. Other recent studies commissioned by *The Lancet* serve to underpin a necessary focus on understanding the determinants of healthy living in Africa [15,16].

In 2012 a new partnership between the University of the Witwatersrand (Wits) and INDEPTH, the AWI-Gen study (Africa Wits-INDEPTH partnership for Genomics studies), was formed. This study is funded by the National Institutes of Health as part of the Human Heredity and Health in Africa Consortium (H3Africa) [17,18]. AWI-Gen’s main purpose is to further understand the prevalence of cardiometabolic disease (CMD), associated risk factors, regional burden, and to explore gene–gene and gene–environment interactions that contribute to disease risk.

**Figure 1.**
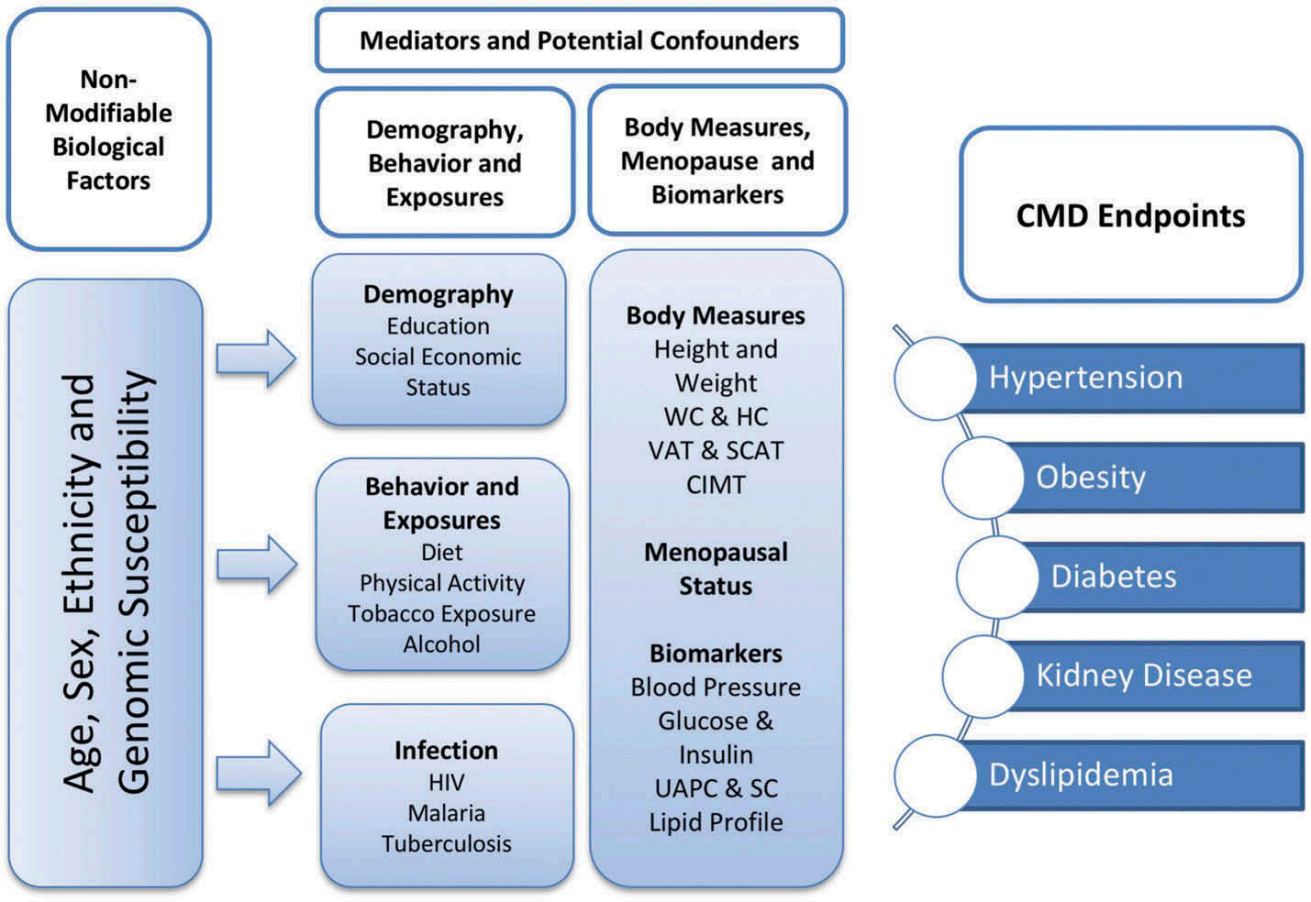
Conceptual framework for Phase 1 of the AWI-Gen study. To begin to understand the cardiometabolic disease endpoints in the six sub-Saharan African communities, we collected data on non-modifiable biological factors and a host of mediators and potential confounders, including behavioural data and infection history and measures of body composition that include height, weight, waist and hip circumference (WC and HC), visceral and subcutaneous fat (VAT and SCAT) and carotid intima-media thickness (CIMT). Also measured were markers indicative of cardiometabolic disease (CMD) including blood pressure, glucose and insulin, urinary albumin protein and creatinine (UAPC) and serum creatinine (SC).

The conceptual framework for AWI-Gen (Figure 1) illustrates the different components of the study that will be explored to increase our understanding of the CMD outcomes in the different regions of SSA. Data on non-modifiable factors such as age, sex and genetic susceptibility to disease, and modifiable factors such as lifestyle, including diet and physical activity, and biomarkers of CMD including BMI, fat distribution, blood pressure and lipid profiles were collected at base-line in Phase 1 of the AWI-Gen study. AWI-Gen deliberately examines older adults aged between 40–60 years, a period during which CMD is most likely to present, and therefore enhances its ability to discover associations between CMD outcomes and risk factors, including genetic susceptibility. In line with the H3Africa mandate, there is a focus on assessing genomic associations with common diseases among Africans [17].

The AWI-Gen partnership leverages unique strengths of the INDEPTH health and demographic surveillance systems network [19] and the MRC/Wits Developmental Pathways for Health Research Unit (DPHRU) in Soweto, Johannesburg [20], to access established frameworks for population-based research. These include infrastructure for longitudinal and cross-sectional field research, experience in establishing community engagement and retention programmes, and facilities in which clinical history, medical examinations, and collection of blood and urine samples can be carried out. The AWI-Gen Collaborative Centre’s central hub at the Sydney Brenner Institute for Molecular Bioscience at the University of the Witwatersrand in Johannesburg, South Africa, also includes a biobank [21] and leads the genomic research with bioinformatics expertise. This, in turn, provides a foundation for training and capacity building for all its partners through a series of workshops and by hosting postgraduate students. AWI-Gen thus provides a research platform for data collection and aggregation with which to study the transitions in African health and disease risk. In our earlier publication [22] we described the AWI-Gen network and our plans to develop a research platform for epidemiology and genomic studies in SSA. In the current paper we describe the detailed methodology for data and sample collection, together with the quality control (QC) processes undertaken during Phase 1 of the AWI-Gen study.

## Methods

### Study setting

The African centres of the INDEPTH network were invited to participate in the study and five Health and Demographic Surveillance System (HDSS) sites responded. Fortuitously they covered West, East and South Africa and represented rural and urban settings. Six study centres in four SSA countries were involved in Phase 1 of the AWI-Gen study: in South Africa, the MRC/Wits Agincourt HDSS [23], Dikgale HDSS [24] and the Soweto centre located within the MRC/Wits Developmental Pathways for Health Research Unit [20]; in Kenya the African Population and Health Research Center HDSS in Nairobi [25]; in Ghana the Navrongo HDSS in the Navrongo Health Research Centre [26]; and in Burkina Faso the Nanoro HDSS hosted by the Institut de Recherche en Sciences de la Santé Clinical Research Unit [27]. The study is coordinated by the AWI-Gen Collaborative Centre based at the Sydney Brenner Institute for Molecular Bioscience, University of the Witwatersrand in Johannesburg, South Africa.

### Study design, participant inclusion and exclusion criteria

The AWI-Gen study [22] is a cross-sectional population study of 10,702 participants aged 40–60 years, recruited between August 2013 and August 2016, plus an additional 1021 participants over the age of 60 recruited at the Agincourt centre. Pregnant women, first-degree relatives of existing participants, recent immigrants (with less than 10 years of residence in the region) and individuals with physical impairments preventing measurement of blood pressure and other anthropometric indices were excluded from the study.

#### Details of sampling frames

Participants were selected via random sampling based on existing population sample frames from the respective research centres. For each of the Dikgale, Nairobi, Nanoro and Navrongo centres, participants were randomly selected until participant recruitment targets of approximately 2000 were attained, comprising similar numbers of women and men. There was some oversampling to account for non-responders and participants who did not meet study inclusion criteria. Various approaches were used in an attempt to remove bias as follows. In Nanoro there was a skewed age pyramid with more women than men, and so the sampling frame was adapted on a village by village basis to include equal numbers of randomly selected women and men. In the Navrongo region there was an uneven distribution of ethnolinguistic groups. To address this, a two-stage sampling frame was used: the west and east zones of the HDSS region were purposefully chosen, and then adults were randomly selected until roughly equal proportions of the two main ethnolinguistic groups (Kassena and Nankana), and a balanced gender ratio had been achieved. In Nairobi, participants were selected using a geographical sampling frame that covered the two peri-urban districts of Korogocho and Viwandani, until roughly equal numbers of female and male participants had been recruited from each district. In Dikgale, participants were selected randomly from a regional geographical sampling frame, but they were unable to reach recruitment targets, especially for men, before the end of the study period in 2016.

Sampling at the Agincourt HDSS in rural Mpumalanga was done from the 2013 population census by identifying all adults aged 40 and above as of 1 July 2014 and who were documented as permanently residing in the study area. Individuals who had participated in previous studies were invited to participate, and the numbers were randomly supplemented up to 2000 in the 40 to 60 year age range, stratified by sex to achieve equal numbers of women and men. Of these, 1465 individuals were successfully recruited into the study. In addition, 1021 individuals over the age of 60 years were recruited for data and sample collection according to the same protocol. Due to migration, the permanent population of the area has a bias in favour of women, and so gender-specific sampling fractions were developed in order to ensure a balance of women and men.

At the Soweto centre, based in Johannesburg, female participants between the ages of 40 and 60 years at the time of data collection were recruited from an existing cohort of caregivers called ‘Birth to 20 plus’ (BT20+) [20]. Approximately 700 of the women selected from this cohort were also part of the ‘Study of Women Entering and Endocrine Transition’ (SWEET) [28]. The remaining participants, including approximately 1000 men, were recruited through random selection using a geographical-based sampling frame that covered the Soweto region.

### Informed consent, ethical considerations and translation of documents

A broad consent process was adopted for the study [29,30] to promote the ethical use of the data and samples for future studies and to protect participants from potential harm. In the rural centres this was preceded by a process of community engagement involving assent from the community leaders.

All participants were provided with an information sheet, ‘Genomic and Environmental Risk Factors for Cardiometabolic Disease in Africans – Trait Association Study’ (see Supplementary Material 1), that described the study. Each individual was then asked to provided written informed consent. These documents were either in English or translated into the local language or dialect of the participant. For those participants who could not read or write, the documents were read in the presence of a witness, and an opportunity was provided to discuss their questions or concerns. It was the responsibility of the trained interviewer to ensure that any questions or objections raised during the consenting process were noted and appropriately addressed. Those who were unable to sign placed their thumb-print on the consent form to denote consent and this was witnessed and signed by a third party. One copy was archived at the local centre and the second given to the participant. The participant was then assigned a randomly generated participant AWI-Gen study ID that was used on all future data related to that participant to ensure protection of the participant’s anonymity. The coded ‘key’ to link participant personal identifying information with the AWI-Gen study ID was kept in a locked cabinet at the local centre and not disclosed to the AWI-Gen Collaborative Centre. All participant consent forms are kept at the individual centres and each centre provided a letter to the Collaborative Centre to confirm that appropriate consent was obtained for all the participants enrolled in the study.

Prospective participants were asked that prior to participating in the study, they refrain from eating or drinking (apart from water) from the night before to enable blood samples to be collected in the fasting state. They were also told that a urine sample would be requested, and were asked to bring any medication which they were currently taking for chronic illness. Participants were provided refreshments after the blood draw, and at some centres a cash reimbursement was provided to cover out-of-pocket expenses incurred by their visit.

Informed consent, information sheets and, in some cases, the questionnaire were translated into local languages when required, by well-trained staff of the centre who were fluent in both languages. The original English questionnaire was then compared to the translated version and discrepancies noted, discussed and resolved. During project training, each question was reviewed by the entire project team and discrepancies noted, discussed, and resolved with consensus. Documents were translated into the following languages: Shangaan (Agincourt), Spedi (Dikgale), Kishwali (Nairobi), French (Nanoro), Kasem and Nankam (Navrongo). No translations were necessary at the Soweto centre.

### Questionnaire design

#### Format

The participant questionnaire was designed using a REDCap (Research Electronic Data Capture) computer database [31,32]. This questionnaire was then transposed to a printable portable document format (PDF) and spacing minimized to generate a compact paper-based questionnaire. This paper questionnaire was then administered to participants at five of the centres. At the sixth centre, Agincourt, the questionnaires were administered in an electronic format using a tablet to facilitate harmonization of the data with another ongoing study – ‘Health and Ageing in Africa: A Longitudinal Study in an INDEPTH Community’ (HAALSI) [33].

#### Data capture and quality control (QC)

The responses to the paper-based questionnaires together with the anthropometry and ultrasound results were manually entered into a REDCap [31,32] computer database installed on a ‘Mac Mini’ computer that was located at each of the centres. Before transferring the data to the AWI-Gen Collaborative Centre, data clerks at each centre checked data for outliers and missing data according to their own internal data management and quality control processes, which included a check of 10% of all entries for consistency between the paper form and the electronic version. Agincourt electronic data was checked by the Agincourt data managers and converted to a form ready for export to REDCap.

Anonymized data were transferred through a secure File Transfer Protocol connection to the central REDCap server at the AWI-Gen Collaborative Centre, where the data was exported into a PostgreSQL database, and a second QC process was performed to search for outliers and missing data, which were then resolved by referring these records back to the local centres for further checks.

At the AWI-Gen Collaborative Centre, a total of 10% of all ultrasound scans were reviewed by a medically trained expert. Intra- and inter-observer differences were estimated by calculating the mean (and 95% confidence interval) of the arithmetic differences between measurements taken in the field and those during QC. The technical error of measurements was maintained at less than the 5% recommended maximum level, with no statistically significant differences observed (*p < *0.05) [34].

Once the data had passed all QC processes, a new version of the database was generated and ‘locked’ to prevent further changes. Data belonging to each centre was then returned for further rounds of QC to check accuracy of data entry and to correct any mistakes or inconsistencies, with each subsequent version of the database being assigned a unique version number and locked to prevent further change.

#### Adaptations of the questionnaire

While the questionnaire was largely identical across centres, some differences were necessary. The section pertaining to ethnicity was adapted according to the local population. The section on household assets was tailored because different assets were relevant to the populations surveyed at the various centres.

The questionnaire for the Soweto site was a modified version previously administered to approximately 700 of the BT20+ women who participated in the SWEET study and excluded questions on home language, education, household assets and marital status. These data were imported from a previous questionnaire to the same participants and entered directly into the Soweto REDCap database. Dietary data using a specific food frequency questionnaire was collected for the women, whilst the dietary behaviour of the Soweto men was collected using the same AWI-Gen questionnaire as used for the other sites.

At the Agincourt centre, an additional 1021 participants over the age of 60 years were recruited to examine CMD in older age. Some of these individuals had participated in earlier studies, and their participation here allowed for longitudinal analysis of CMD risk factors [35,36]. The questionnaire administered to participants only included questions not already included in the HAALSI study. A limited subset of the measures used for Phase 1 of the AWI-Gen study were collected for these participants. For those participants over the age of 70 years, the data and blood samples were collected at the participant’s home by a phlebotomist, and not at the Agincourt clinic, and therefore no ultrasound measurements were collected for this older group.

#### Questionnaire domains

The questionnaire (see Supplementary Material 2) requested information on demography, family composition, family ethnicity, reproductive history (females), marital status, education, employment, household attributes, diet, substance use (tobacco, alcohol, and drugs), general health, exposure to pesticides, infection history (HIV, TB, malaria), history of CMD (diabetes, stroke, hypertension, angina, heart attack, congestive heart failure, obesity, high cholesterol), thyroid disease, kidney disease, physical activity and sleep. The questionnaire took approximately one hour to complete.

### The questionnaire contents

#### Demographic factors and socioeconomic status

Self-identified home language and ethnicity with which the participant best identified was recorded. Participants were also asked about the home language and ethnicity of their parents and grandparents. Marital status included six options to enable categorization as being married, cohabiting with partner or being single (which included being unmarried, divorced, separated or partner deceased).

Education was probed by asking about the highest level of education (no formal education, primary, secondary or tertiary education), and how many years were completed at the highest education level.

Employment status was captured by asking the participants whether they were employed (including part-time employment and self-employment) or unemployed.

Household density was defined as the number of people living in a given household divided by the number of bedrooms in the house. In the case of households of families that live in ‘compounds’, the household density was calculated as the total number of people living in the compound divided by the total number of bedrooms in the compound.

Socioeconomic status (SES) was estimated by calculations based on household assets, according to the practice implemented by the Demographic and Health Surveys (DHS) Program [37,38]. The method involves a principal component analysis of the SES variables (household assets), predicting factor scores, and then categorizing these scores into quintiles for each of the centres separately. The quintiles were then used to do within-centre and cross-centre comparisons.

#### Reproductive history and menopausal status

Participants were asked to provide details of the number of siblings with whom they share at least one parent and their number of biological children.

Menopause, staged according to the guidelines of the North American Menopause Society [39], was limited to observation of the participant’s menstrual cycle. Menopause status was categorized as follows: pre-menopausal as having current regular periods; peri-menopausal (i.e. menopausal transition) as having irregular periods within the past 12 months; post-menopausal as having no period within the past 12 months. At the Soweto site, participants were asked if they were taking contraceptives, or had a hysterectomy, in which case their menopause stage was labelled as indeterminant.

#### Behavioural factors

Dietary factors that represent key behaviours related to lifestyle were explored using a series of questions guided by the WHO Steps Instrument [40] about the consumption of fruit and vegetables (supported with show cards to indicate portion size), vendor-supplied meals, bread, and sugar-sweetened drinks including fruit juice.

The Global Physical Activity Questionnaire (GPAQ) was used to obtain self-reported physical activity [41]. Total moderate-vigorous physical activity (MVPA) in minutes per week was calculated from the accumulation of occupation, travel-related and leisure time physical activity. Sitting time (minutes/week) is used as a proxy for sedentary behaviour. Participants were classified as active if their MVPA was ≥ 150 minutes/week or inactive if their MVPA was < 150 minutes/week.

Drinking was categorized into: never consumed; current non-problematic consumer; current problematic consumer; former consumer. Problematic drinking of alcohol was determined according to the CAGE questionnaire [42], where four questions related to potential problematic alcohol consumption were asked, and categorized as problematic if the participant answered ‘yes’ to at least two of them.

The consumption of smoked tobacco products, and smokeless tobacco such as chewing tobacco and/or snuff tobacco, were each categorized as follows: never used; current user; former user. The number of cigarettes or times a tobacco product were consumed daily was recorded [43,44].

#### Pesticide exposure

Participants were asked about insecticide/pesticide exposure in their work environment and duration of exposure, use in their homes or in their local area. Lifetime exposure to pesticides was determined as the number of years of pesticide exposure [45].

#### Infection history

Participants were asked if they ever had malaria and whether they have had malaria fever in the past month. They were also asked about tuberculosis, time of diagnosis and treatment.

HIV status was self-reported as positive or negative. Participants from Kenya and South Africa were offered a voluntary HIV rapid test (using a locally available, government-approved rapid-test). Participants at the Burkina Faso and Ghana sites were not offered HIV tests, which was a decision based on the low prevalence of HIV in these countries, and the potential that stigma in the local communities against people living with HIV may cause emotional harm [46]. Importantly, nobody was excluded from this study based on their HIV status, or if they chose not to be tested.

#### Physical (anthropometric) measurements

Standing height to the nearest millimetre was measured using a Harpenden digital stadiometer (Holtain, Wales) fixed to a wall. Participants were either barefoot or wearing thin socks. Where possible heels, buttocks, shoulders and the back of the head were touching either the wall or the vertical board of the stadiometer. If this was not possible, a straight posture was maintained with at least the buttocks and heels touching the vertical board. Heels were positioned together with the medial borders of the feet forming a maximum angle of 60°, shoulders relaxed but straightened, and arms hanging freely alongside the body with palms facing inwards. The head was positioned in the Frankfurt Horizontal Plane and the participant asked to take a deep breath and maintain an erect posture. The headboard was brought down onto the vertex of the head with sufficient pressure applied to compress the hair, and care taken not to push the subject down.

Weight was measured to the nearest 0.1 kg using digital Physician Large Dial 200 kg capacity scales (Kendon Medical, South Africa). Participants were asked to remove shoes, heavy clothing and jewellery, and to empty their pockets prior to mounting the centre of the platform, with a 10 cm gap between their heels, leaving arms hanging loosely at their sides.

Waist circumference was measured using a stretch-resistant tape measure (SECA, Hamburg, Germany). Participants were asked to wear only light clothing, with outer clothing removed to enable the tape to be positioned correctly; with the participant standing up straight with arms slightly abducted, and breathing normally, the tape was placed horizontally around the narrowest part of the torso, about halfway between the iliac crest and the lowest rib. Measurement of waist circumference was taken at the end of a normal expiration. Waist circumference was recorded in millimetre. The same tape was used to measure hip circumference, with the tape placed around the most protruding part of the buttocks, ensuring that the zero mark was to the participant’s side. Hip circumference was recorded to the nearest millimetre.

All physical measurements were taken by an experienced research nurse or trained research assistant at the centre. Measurement equipment was purchased centrally to ensure standardization. All equipment was calibrated to the required standards and maintained across all centres. Additionally, for the Ghana site, the Ghana Standards Authority, as a statutory requirement, inspected all equipment to ensure that they were calibrated to prescribed standards. There were subsequent yearly inspections and when necessary recalibration was done. The process of measuring height, weight, and waist and hip circumferences took a maximum of 15 minutes.

#### Measurement of blood pressure

Blood pressure was measured using a digital sphygmomanometer (Omron M6, Omron, Kyoto, Japan). Participants were seated upright with their back supported and feet firmly resting on the floor and not crossed. To ensure that the antecubital fossa was at the level of the heart, participants were seated with the arm resting on a desk or arm rest, with palm facing up. An appropriately sized cuff was placed on the arm about 2 to 3 cm above the antecubital fossa, and not restricted by clothing. The participant was asked to relax for three to five minutes before systolic blood pressure (SBP), diastolic blood pressure (DBP) and pulse rate were measured. The time that the measurement was taken was recorded. Blood pressure and pulse rate measurements were repeated two more times at two-minute intervals. The mean of the last two measurements was used to calculate the final average systolic and diastolic blood pressure results and pulse rate. All measurements were taken by a trained healthcare professional. Participants were instructed to abstain from heavy exercise and alcohol consumption the day prior to and on the day of their appointment at the clinic as these could affect the measurements.

#### Ultrasound measurements

A LOGIQ e ultrasound system (GE Healthcare, CT, USA) was used to measure visceral (VAT) and subcutaneous adipose tissue (SCAT), as well as carotid intima-media thickness (CIMT). A 2–5 MHz 3C-RS curved array transducer was used to measure both VAT and SCAT while a 12L-RS straight transducer was used to measure CIMT. To ensure consistency across all centres, selected researchers (including nurses, clinicians and researchers) from each site were trained centrally at the DPHRU in Soweto. The ultrasound examinations took approximately 30 minutes to complete and images and semi-automated measurements were recorded on a sample data collection sheet and transcribed to the paper questionnaire. Images were saved on a hard drive to prevent loss of data after examination, and periodically bulk-uploaded or transferred using a hard drive to the AWI-Gen Collaborative Centre server for archiving.

To capture VAT measurements, the ultrasound machine measurement was set to a depth of 15 cm and image to be captured labelled as ‘medial’. With the participant in the supine position, gel was applied to the lower abdomen and the probe positioned with minimal compression on the midline at a level midway between the lower costal margin and the iliac crest. The xiphi-sternum and umbilicus were used as a guide for accurate positioning. The spine was positioned horizontally, and the vertebra centrally in the image, and the gain setting adjusted accordingly. The participant was then asked to breathe quietly, and the measurement was taken at the end of the expiration. To calculate the amount of VAT, the paused ultrasound image was brought up onto the screen. The first cursor was placed anterior to the spine (on the fat pad if visible), and the second cursor on the thin peritoneal layer beneath the anterior rectus abdominus muscles. Care was taken to ensure that the measurement was perpendicular to the surface of the lumbar vertebra, and taken between the peritoneum and the spine, where there is a clear space between the vertebra and the aorta [47,48]. The measurement was repeated by producing a second image, and the results recorded in centimetres to two decimal places on a Sample Collection Data Sheet. The researcher did an immediate quality check to ensure that the spine, abdominal aorta and rectus abdominus muscle were visualized on the image.

The measurement of SCAT (transverse) was performed directly after the medial measurement to ensure that the image was saved under the same unique identifier assigned to the participant. However, the image was immediately relabelled accordingly as ‘subcut’. The ultrasound probe was rotated through 90 degrees and depth setting adjusted to 9 cm. The rectus abdominus muscles were visualized, with care taken to ensure that both muscles were symmetrical in the image, and that the linea alba was centrally located, the gain adjusted accordingly, and the image captured. To calculate the SCAT thickness, the distance between the skin and the outer edge of the linea alba was measured on the screen, as described above. The measurement was then taken from a second image, and the results recorded similarly, in centimetres to two decimal places.

To measure the right carotid intima-media thickness (CIMT), the participant was asked to lie down in a supine position with a pillow underneath the neck for slight extension, head turned towards the left and gel applied to the exposed neck area. Using the two sternocleidomastoid muscles as landmarks, the exposed area was scanned along the longitudinal plane until the common carotid artery (CCA) was found, and an image taken. The CIMT of the far wall (posterior) of both the left and the right CCA was measured for this study. The CIMT was calculated by placing a cursor at two points on the posterior wall of an approximately 10 mm segment of the artery. The proximal starting point was 1 cm from the bulb of the CCA [49,50]. The instrument software automatically captures the distance of the intima–media interface as the CIMT measurement. Measurements were taken of minimum, maximum and average distance in centimetres to two decimal places. To measure the left carotid, the participant turned on to the opposite side, and the process was repeated. Data were interpreted according to the Mannheim Consensus [51].

### Blood and urine sample collection, processing, analysis and storage

Fasting venous blood samples were collected by a trained phlebotomist according to the WHO standards of best practice [52]. Participants were asked not to eat or drink after their evening meal of the previous day to ensure that a fasting blood sample was taken. Collection tubes were labelled with a pre-printed AWI-Gen barcode. Two 6 ml clot activator tubes (red capped), one 4 ml potassium oxalate tube (grey) and two 6 ml potassium-EDTA (purple capped) tubes were collected from each participant, equivalent to approximately 28 ml of blood in total. After the blood draw, potassium oxalate and potassium EDTA tubes were inverted to ensure mixing of the anticoagulant and left to stand at room temperature for 15–30 minutes before processing. Serum and plasma were separated using a centrifuge with swing buckets at 1500–2000 x g (3000 rpm) for 10 minutes at room temperature. The supernatant was aliquoted into 1 ml samples in 2 ml cryovials labelled with pre-printed AWI-Gen barcodes for each participant and stored at −80°C until shipping on dry ice.

To obtain buffy coats, potassium EDTA tubes were centrifuged at 1500–2000 x g for 15 minutes at room temperature, the plasma (approximately 2 ml) removed and buffy coat (approximately 500 μl) lifted and aliquoted into individual 2 ml tubes and stored as above.

Midstream urine samples were collected in accordance with the procedures followed by the WHO [53]. The genital region was cleaned with an antiseptic pad, the first volume of urine voided into the toilet, and the midstream volume of 10–15 ml voided into a sterile collection pot. Samples were centrifuged using a swing bucket rotor at 1500–2000 x g for 5 minutes, the supernatant aliquoted into four cryovials of 2 ml each. Samples were stored at −80°C until shipping.

Serum, plasma, buffy coat, and urine samples were shipped on dry ice to the AWI-Gen Collaborative Centre. Upon arrival, they were transferred to a −80°C freezer for storage prior to analysis of biomarkers, and extraction of genomic DNA.

### Biomarker analyses

All analyses were performed according to good laboratory practice, and with external monitoring for quality control. Fasting blood (serum and plasma) biomarker assays were performed at the laboratory of the DPHRU in Soweto under the guidance of a pathologist. Urine biomarkers were measured at Clinical Laboratory Services in Johannesburg, South Africa (CLS, a company of the University of the Witwatersrand). To avoid potential batch effects, samples from different centres were batched across different analysis runs with appropriate standard controls.

Serum glucose, lipids and creatinine were analysed using a Randox Plus clinical chemistry analyser (UK) using colorimetric assays. The Randox Glucose assay has a wide measuring range of 0.36–35 mmol/l. The concentrations for triglycerides, total cholesterol, and high density lipoprotein cholesterol (HDL-C) were determined directly from the assay, with measuring ranges of 0.13–5.60 mmol/l, 0.30–17.20 mmol/l and 0.05–3.80 mmol/l respectively. The concentration of low density lipoprotein cholesterol (LDL-C) was measured directly with a measuring range 0.40–4.90 mmol/l, but was also calculated indirectly using the Friedewald equation [54]. Non-HDL-C was calculated by subtracting HDL-C from the total cholesterol [55]. The measuring range of creatinine was 11.80–2448 μmol/l. The coefficient of variation of the laboratory measurements for glucose, lipids and creatinine were less than 2.3%, 1.5% and 0.8%, respectively.

Fasting serum insulin concentrations were determined using the Immulite 1000 chemistry analysis system (Siemens, Germany). Insulin concentrations were determined by a solid-phase, enzyme-labelled chemiluminescent immunometric assay. The measuring range was 2–300 μIU/ml. The coefficient of variation for the measurement of this biomarker was less than 2%.

Urine biomarker assays were performed by using a Roche/Hitachi Cobas C501 System. Urine creatinine was measured using a kinetic colorimetric based assay with a measuring range for the assay of 3.75–550 mmol/l. The laboratory coefficient of variation was less than 2.8%. Microalbumin and total protein concentrations in urine samples were determined using a turbidimetric assay. The measuring range was 3–400 mg/l for microalbumin, and the coefficient of variation was less than 2.7%. For total protein, the measuring range for this assay was 10–1000 mg/l, with a coefficient of variation less than 3%.

### Isolation and purification of genomic DNA

Isolation and purification of genomic DNA was performed using automated or manual DNA extraction protocols as follows. Genomic DNA was purified from buffy coats using the automated QIASymphony SP (Qiagen, Germany) sample purification robot and the Qiagen magnetic bead-based DNA mini-prep kit (catalogue number 931,255) running Qiagen protocol DNA_BC_400_V6_DSP and the genomic DNA eluted in a final volume of 400 μl. DNA was quantified using a Nanodrop spectrophotometer (Thermo Lifesciences, USA) or a Qubit fluorimeter using the Qubit dsDNA BR Assay Kit (Thermo Life Sciences, USA). Manual extraction of genomic DNA was performed according to a modified salting out procedure [56]. Essentially, nucleated cells from buffy coats were resuspended in 3 ml of 10 mM Tris-HCl, 400 mM NaCl, 2 mM Na_2_EDTA buffer in a 15 ml Falcon tube and digested overnight at 37°C by proteinase K (1 mg in 0.5 ml 20 mM Na_2_EDTA). After the addition of 1 ml saturated (6 M) NaCl and shaking, the protein precipitate was removed by centrifugation and the DNA-containing supernatant transferred to a fresh falcon tube. Two volumes of ethanol were added to the supernatant, causing the DNA to precipitate as cloudy strands in the solution, which were transferred to a 1.5 ml microtube using a tooth pick, and dissolved in 100–200 μl of TE buffer (10 mM Tris-HCl, 0.2 mM Na_2_EDTA, pH 7.5) for 2 hours at 37°C. Samples were divided into 4 aliquots and stored in 2D barcoded cryotubes. Two of these aliquots were retained at the SBIMB Biobank, one sent to the H3Africa Biobank in Johannesburg, and the final aliquot returned to the local centre from where the samples originated. To avoid repeat freeze/thaw cycles, the SBIMB Biobank stored one of the aliquots at 4°C, and all other samples were stored at −80°C.

Three methods were used for quality control of the genomic DNA: agarose gel electrophoresis and spectrophotometry for all samples, and limited sexing by PCR. To examine the integrity of the genomic DNA, 1 μl of the DNA was loaded onto a 0.8% agarose gel and electrophoresed at 100 V for 1 hour in the presence of 0.2 μg/ml ethidium bromide. DNA was visualized under UV light. Each sample was viewed to check that there was a single band of high molecular weight genomic DNA, and the absence of a smear that would indicate DNA fragmentation, degradation or salt contamination. Spectrophotometry was used to assess potential protein contamination by calculating the OD 260 nm/280 nm ratio. Values were consistently between 1.8 and 2.0 indicating that purification of the genomic DNA was effective. PCR was used to determine the sex of a limited number of participants in the QC batches. This provided a mechanism to confirm that DNA was suitable for downstream PCR-based analyses, as well as a cross-check to confirm that reported sex matched sexing results using a method adapted from previously published work [57,58]. Essentially, PCR is used to amplify two genes simultaneously. The first is specific for the amelogenin gene (forward primer 5ʹ-CTG ATG GTT GGC CTC AAG CCT GTG-3ʹ; reverse primer 5ʹ-TAA AGA GAT TCA TTA ACT TGA CTG-3ʹ), generating chromosome specific fragments sized 927 base pairs for the X chromosome and 738 base pairs for the Y. The second PCR assay is specific for the *SRY* gene (forward primer 5ʹ-CGA CAA TGC AAT CAT ATG CTT CTG-3ʹ; reverse primer 5ʹ-CTG TAG CGG TCC CGT TGC TGC-3ʹ) present only on the male-specific Y chromosome, generating a product sized 600 base pairs. After gel electrophoresis (on an 2% agarose gel as above) samples from male participants yielded three fragments, and females just one.

### Classification of CMD disease outcomes

The CMD disease outcomes, hypertension, obesity, diabetes, kidney disease and dyslipidemia, were categorized as below.

#### Hypertension

Classification of hypertension was guided by the JNC7 guidelines of the Joint National Committee of Prevention, Detection, Evaluation, and Treatment of High Blood Pressure [59]. As such, hypertension was defined as the presentation of one or more of the following conditions: previous diagnosis by a healthcare provider (which excluded cases of hypertension during pregnancy), taking medication for the condition, systolic blood pressure greater than 140 mm Hg and/or diastolic blood pressure greater than 90 mm Hg at the time of the study visit [60].

#### Obesity

Body Mass Index (BMI, kg/m^2^) was calculated as both a continuous variable, and categorized according to the standards adopted by the WHO [61], using the following cutoffs: underweight (BMI < 18.5 kg/m^2^), normal weight (BMI ≥ 18.5 and < 25kg/m^2^), overweight (BMI ≥ 25 and < 29.9 kg/m^2^) and obese (BMI ≥ 30 kg/m^2^).

Waist-to-hip ratios were derived by dividing the waist circumference (mm) by the hip circumference (mm), and the results interpreted in accordance with the standards adopted by the WHO [62]. Substantially increased risk of metabolic complications were judged as high if the waist-to-hip ratio (WHR) was ≥ 0.9 for men, and ≥ 0.85 for women.

#### Diabetes

Classification of diabetes was guided by the standards set by the American Diabetes Association [63]. It was defined as the presentation of one or more of the following conditions: previous diagnosis by a healthcare provider (which excluded gestational diabetes), taking medication for the condition, or a fasting blood glucose level of ≥ 7.0 mmol/l. Participants were defined as pre-diabetic if the levels of fasting blood glucose were between 5.6 mmol/l and 7.0 mmol/l.

#### Kidney disease

As an indication of the presence and stage of kidney disease, we assessed the urine albumin/creatinine ratio (ACR) as mg albumin/mmol creatinine [64]. For men, normal was < 2.5, early stage kidney damage (microalbuminuria) 2.5–25, late stage kidney damage (macroalbuminuria) > 25. For women, normal was < 3.5, early stage kidney damage (microalbinuria) 3.5–35, late stage kidney damage (macroalbuminuria) > 35. Calculated eGFR was used as supporting evidence of kidney disease and will be reported in a separate paper.

#### Dyslipidemia

Dyslipidemia was defined as the presence of one of the following indications: self-reported history of high cholesterol, previous diagnosis of dyslipidemia by a healthcare provider, taking medication for the condition, measured total cholesterol level ≥ 5.0 mmol/l, LDL-C ≥ 3.0 mmol/l, and HDL-C < 1.0 mmol/l for men and < 1.3 mmol/l for women, or triglycerides > 1.7 mmol/l, and non-HDL-C > 3.4 mmol/l [65]. These data will be reported in a separate paper.

### Data and sample storage

The AWI-Gen data and samples will be shared with the global scientific community according to the informed consent provided by the participants, in line with the ethics approvals and according to the H3Africa policies and guidelines (www.h3africa.org). In addition to access through direct collaboration, applications can be made to access the data through the European Genome Phenome Archive (EGA) and the samples through an H3Africa Biobank, by applying to the H3Africa Data and Biospecimen Access Committee.

## Results

### Age and country distributions of the AWI-Gen participants

**Figure 2.**
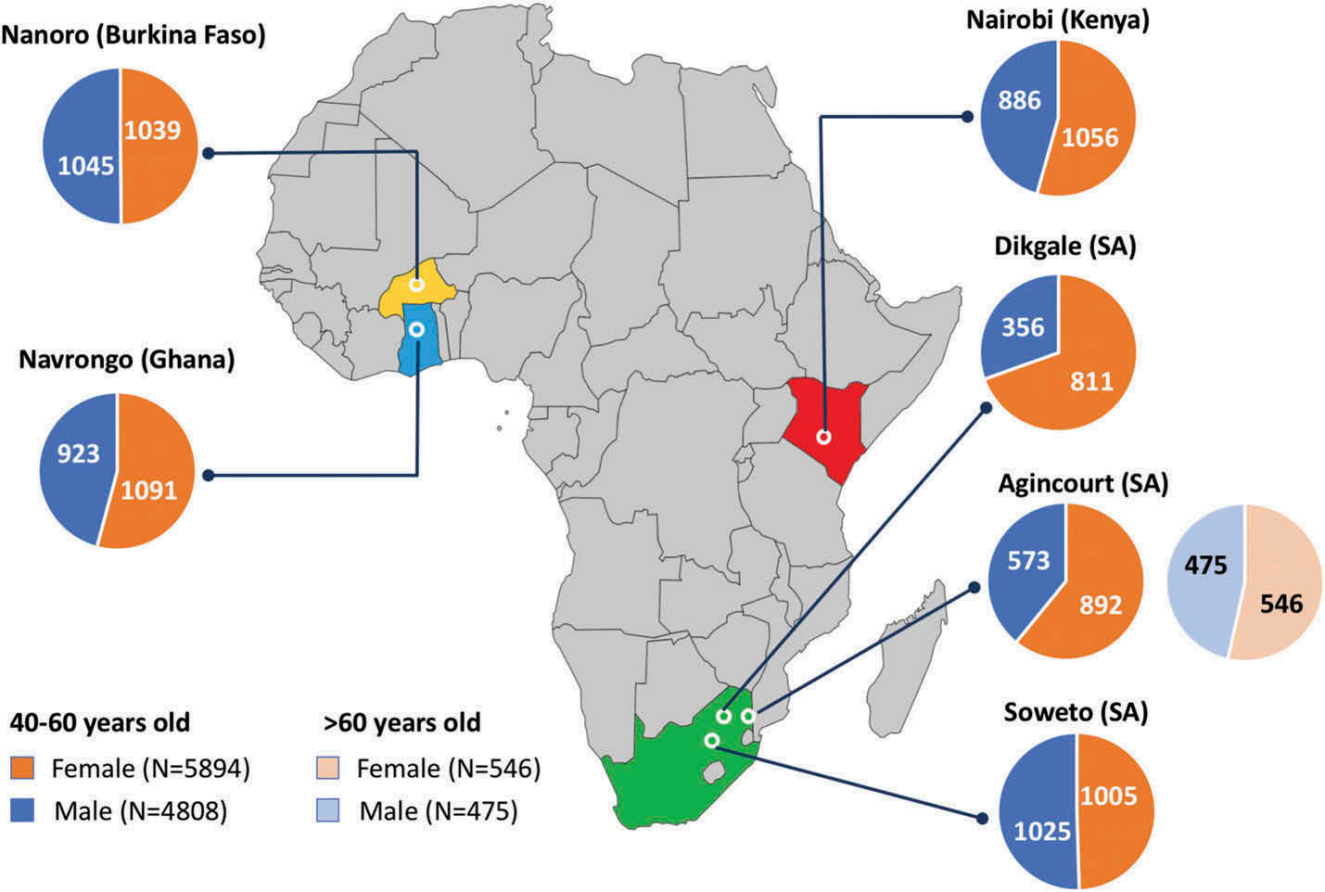
Distribution of the AWI-Gen study participants across the six centres located in Nanoro (Burkina Faso), Navrongo (Ghana), Nairobi (Kenya), and Dikgale, Agincourt and Soweto in South Africa (SA). The pie charts represent the total numbers of female (orange segments) and male (blue segments) participants aged 40–60 years for each centre, and participants over the age of 60 years for Agincourt.

**Figure 3.**
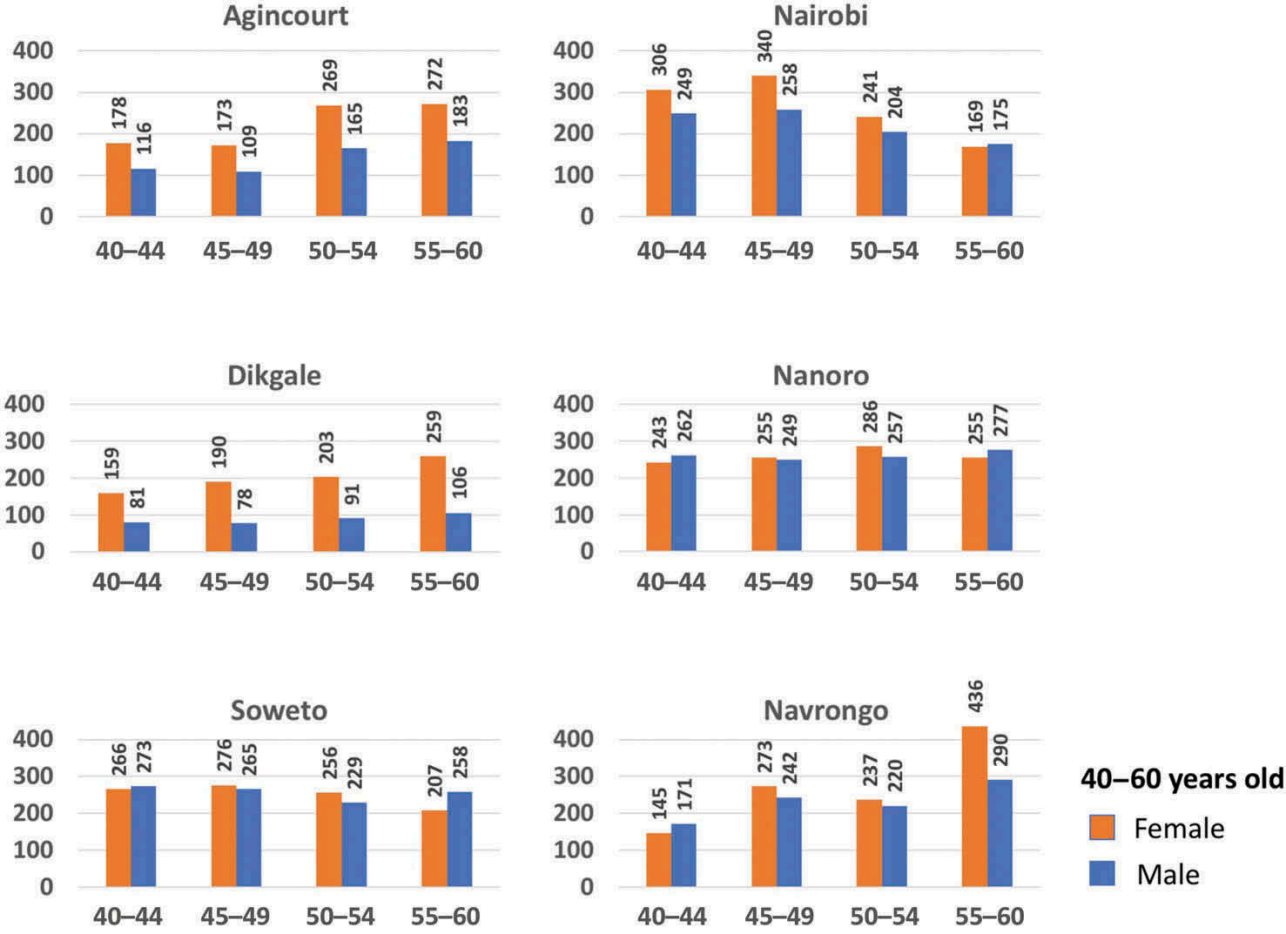
Distribution of female (orange bars) and male (blue bars) study participants aged between 40 and 60 years across the six AWI-Gen centres, stratified according to age group 40–44, 45–49, 50–54 and 55–60 years.

During the period from 2012 to 2016, a total of 10,702 participants between the ages of 40 to 60 years were enrolled across the six AWI-Gen centres, and a further 1021 over the age of 60 enrolled at the Agincourt centre. Following community engagement and individual informed consent, data and samples were collected according to the procedures described above. Age and gender of participants, stratified by participating research centre, are shown in Figure 2, and stratified according to age groups (40–44, 45–49, 50–55 and 55–60 years) in Figure 3.

### Challenges and how we addressed them

Developing and implementing a complex multi-regional epidemiological and genomic study across SSA presented a series of challenges. These are grouped under the four categories described below.

#### Limited ethical and legal frameworks for genomic biobanking research

To address the absence of legal and ethical frameworks and guidelines for large-scale genomics studies in the African countries where the AWI-Gen study is taking place, we convened a workshop in December 2012 for members of ethics committees from the four countries involved. We specifically addressed four areas of concern: community engagement and information sharing; informed and broad consent; sample and data sharing; and benefit sharing [29]. This workshop was important in highlighting the need for further discussion and education of committee members about genomic and biobanking research. It was important in the facilitation of the ethics review process for the AWI-Gen centres to inform and support ethics applications to each of the institutional or national review committees for the AWI-Gen centres in Burkina Faso, Ghana, Kenya and South Africa. It should be noted, however, that the time taken to approach and receive approval from the committees was substantial, and delayed entry to the field by approximately a year.

We worked in concert with the Human Research Ethics Committee (Medical) and the sub-committee for Biobanking Ethics of the University of the Witwatersrand to develop a framework within which our study could operate. This resulted in the development and approval of the SBIMB Biobank that operates according to international norms and standards and is described in a publication providing guidelines on establishing a biobank in a resource-poor environment [21].

Members of the AWI-Gen group provided significant input from personal experience toward the development of H3Africa Guidelines for community engagement and informed consent (www.h3africa.org). Our protocols were included in an analysis of H3Africa Consortium consent documents [66] and we contributed to discussions on promoting fairness for genomic research in an African context [30].

#### Data collection and QC

The use of paper-based questionnaire instruments brings a series of operational challenges. Data clerks need to transcribe the information into electronic form, which then needed to be double checked for errors. At the time there were limited options available to the study, as centres lacked the computer infrastructure, internet connectivity and the system administration support required for electronic capture. With recent advances in the development of REDCap, and simplified network administration tools using LINUX operating systems, there is now no real justification to remain with paper-based tools, except for use as a backup in the case of computer failure.

#### Achieving recruitment targets

Overall, the study sites did a remarkable job of recruiting the required participants according to the inclusion criteria (Figures 2 and 3). One of the AWI-Gen centres, Dikgale in South Africa, failed to reach their recruitment target of 2000 participants between the ages of 40 and 60 years, comprised of roughly equal numbers of women and men. This was because many men of working age temporarily move to the urban regions to seek jobs. They then either live far away for extended periods or only return home on weekends and are then reluctant to spend a day participating in a research project. Of the 1165 participants recruited, ~ 70% were female. The challenge was only realized late in the study, and it was not possible to extend the window for recruitment further. The Agincourt site recruited 1465 participants between the ages of 40 and 60 years with ~ 60% females, and recruited an additional 1021 participants over the age of 60 years, with ~ 53% being female.

#### Blood draw

At several centres, a number of participants expressed dismay and discomfort at the number of tubes of blood that were taken. During community engagement meetings with local chiefs, we learnt that it is very important to carefully explain exactly what each tube of blood would be used for, and to work with the local community leaders to help quell any rumours that the procedure was bad for your health.

### Feedback of biomarker test results

Although those with high blood pressure were referred to their local healthcare providers, we did not do any point of care testing. At the end of Phase 1 of the AWI-Gen study, feedback of potentially clinically relevant information was provided to participants, but since the time lapse between the fasting venous blood collection and the laboratory results was several years, the results were not of health benefit. If fasting glucose or lipid levels were of concern, the data feedback was accompanied by a recommendation to follow up with a local healthcare practitioner. BMI was reported and all participants were encouraged to practise a healthy lifestyle in terms of diet and exercise.

## Discussion

### Building a baseline to describe CMD in SSA

Collection of health data across communities and regions in SSA is key to developing an understanding of the diversity and patterns of NCD burden. However, the differing languages and cultures, varying norms and practices of healthy living, hugely disparate levels of poverty, and the heterogeneity between rural and urban communities make direct comparison of findings a challenge. To begin to explore and understand the interacting factors related to CMD risk and its endpoints, we collected demographic, health, wealth and behavioural data and biomarkers in six African communities that span East, West and Southern Africa. The breadth of data we have obtained will allow us to determine associations with disease and modifiable factors such as BMI, fat distribution and physical activity, together with biomarkers of disease and disease endpoints.

The importance of the baseline data generated from this study has been demonstrated in several recent publications, including the identification of regional and sex differences in the prevalence of hypertension across SSA [60], and the six papers on BMI distribution in this special edition of *Global Health Action* [67–72]. Manuscripts discussing the prevalence of diabetes, body composition and obesity, dyslipidemia, kidney disease, and smoking and alcohol consumption are currently in preparation.

The methods described here take into consideration other CMD cohort studies, providing an opportunity for further harmonization with other studies, and for meta-analyses examining specific research questions expanding over multiple studies. At the outset, the variables were harmonized with the HAALSI study [33], but they have now also been transformed for equivalence with the Cardiovascular H3Africa Innovation Resource (CHAIR) which includes approximately 53,000 individuals [73].

### The need for an engaged community

Planning research that is conducted in a respectful manner, and which reaps maximum social value, is one of the fundamental goals of community engagement [29]. In this study, centres reached out to communities to discuss the nature of the research, how confidentiality would be maintained, and how individual rights as a participant would be protected. One major challenge with genomics research is that scientific language can be abstract and difficult to understand [74]. This meant that field staff had to develop verbal constructs to explain, for example, what genes are and how they are inherited, and to be able to distinguish between diseases that are monogenic or are polygenic and multifactorial in nature. An important concern was that the study might introduce therapeutic and diagnostic expectations [75,76]. Participants were informed that if there were clear clinical indications of disease, then they would be referred to a local medical practitioner or health facility for treatment according to local standards of care. As the nature of diseases being studied by AWI-Gen are primarily polygenic and multifactorial in nature, disclosure of genome-wide association studies (GWAS) and/or whole genome sequencing results would not benefit the individual, and therefore it was made clear to participants that they would not receive individual genetic results. This would include incidental findings of known pathogenic variants. Since our participants are older adults, early onset monogenic traits would already have manifested clinically and would not be detected as new conditions in this study. This being said, if monogenic disease-causing variants for which there is effective treatment are found in the future, then the situation would be addressed in close consultation with the H3Africa ethics advisory committee, and the respective ethics review committees for AWI-Gen and the field research centres.

### A requirement for broad consent, data storage and biobanking

In their paper examining broad consent for genomic research and biobanking in low- and middle-income countries [77], Tindana and de Vries reviewed whether it was morally acceptable to ask that participants provide samples for broad use and sharing within the scientific community. They found evidence to support earlier work that indicated that this could be justified provided that researchers were trustworthy, the research was accompanied by community engagement, and that appropriate governance structures were in place [78]. They also identified a concern that revisiting participants for reconsent for each new study was impractical. In the AWI-Gen study, participants were asked to provide broad consent, under the condition that strict regulatory conditions were in place, and any new studies of the participant samples would need to be approved by an ethics review board. The participant has the right to request removal from the study at any time. This would entail the destruction of specimens held in each of the biobanks and no additional data being generated. It would not be possible to remove data from databases once it had been submitted to an international data repository such as the European Genome-phenome Archive (EGA).

### Limitations of the study instruments

In a research setting, it is not practical to diagnose hypertension and diabetes using clinical standards of repeated measurements, as specified in the JNC7 and ADA guidelines [59,63]. This is acknowledged as a limitation of the study. Any participants who presented evidence of high blood pressure were referred to their local healthcare provider for further examination.

Psychosocial issues, respiratory health, and frailty in ageing populations are increasingly recognized as important co-variates of CVD, and were not adequately addressed in Phase 1 of the AWI-Gen study. In Phase 2, we aim to collect baseline data to redress some of these issues.

According to recent surveys, the prevalence of HIV infection in Burkina Faso (Nanoro) and Ghana (Navrongo) is low (approximately 0.7% and 1.0% respectively [79–81]), and so, given the size of study populations, we do not expect that analysis of HIV as a confounder of CMD risk will be statistically significant for these regions.

## Future perspectives

### The trove of genomic data

Phase 1 of the AWI-Gen study includes GWAS to analyse all participant DNA samples using the Infinium H3Africa SNP array (Illumina, USA). The H3Africa SNP array contains approximately 2.39 million SNPs and will be described in detail in a separate publication. Whole genome sequencing data will be available for approximately 160 AWI-Gen participants from West and South Africa. These individuals were chosen because they are from under-represented ethnolinguistic groups in global data sets and will therefore make an important contribution to reference panels that will be used for genotype augmentation using imputation techniques. The genome data from AWI-Gen will enable one of the first SSA-wide studies of the genetics of CMD and associated risk factors and, being a cross-sectional study, will also provide an ideal population-based control for other genomics studies, including disease-related case-control studies in Africa. A recent perspective piece in *Nature Genetics* highlighted the importance of cohort studies linked to GWAS data for establishing causality and developing new therapeutic approaches [82]. This is especially important for African cohorts that are scarce, yet have the unique potential for the discovery of novel disease-related associations.

### Phase 2 of the AWI-Gen study

This study was conducted in the context of the H3Africa Consortium, and provides opportunities for developing large African datasets with harmonized phenotype and genetic data to provide improved power for discovery as well as data for replication studies and meta-analyses.

AWI-Gen itself provides a rich and complex dataset and sample resource for studying CMD in four African countries. By revisiting participants of Phase 1 of the AWI-Gen study three to four years after the first visit, we will develop a set of longitudinal cohorts that will follow the evolving health burden and progression of cardiometabolic disease trajectories. This will be facilitated by continuing to leverage the excellent community relationships established and maintained through the INDEPTH Health and Demographic surveillance centres and the Soweto Developmental Pathways for Health Research Unit.

In Phase 2 of the AWI-Gen study we will make several key changes. The first will be to leverage electronic capture systems for questionnaire data. This will streamline data collection and provide for online ongoing QC of data, and reduce the time taken from collection to data release. The second change will be the introduction of point of care testing (PoCT) for glucose and cholesterol. Our aim here is to provide immediate feedback to participants and to improve our contribution to public health initiatives, which in turn will improve the levels of engagement with participant communities. While the biomarker data we collect is for research purposes only, the ability to provide some clinical benefit through PoCT for participation in the study brings more purpose and value to those who volunteer their time to us. Third, our questionnaires will expand to include the domains of psychosocial factors, respiratory health, and issues of frailty, understanding that these have an important impact on CMD outcomes in ageing populations. Finally, we will extend the study to consider two additional dimensions of CMD: the hormonal and epigenetic changes that occur over the menopause, and the interplay between CMD and the microbiome.

### Data and sample sharing and access

The H3Africa Data and Biospecimen Access Committee (DBAC) can be approached to request access to data in EGA and DNA samples stored in an H3Africa Biorepository. We would also welcome direct requests for collaboration.

## Conclusions

This paper describes the rigorous standard operating procedures and cross-centre harmonization performed in collecting the data for the first phase of the AWI-Gen Collaborative Centre study.

There are several key aspects that have led to the success of the AWI-Gen study. They include the experience of the INDEPTH research network and the AWI-Gen team, its attention to community engagement, and the development of processes for broad consent and storage of data and specimens, including DNA, for future use. The study had its challenges. Initially these were related to establishing acceptable norms and obtaining ethics approval for such a large-scale genomics study. Then secondly, the need for appropriate cultural adaptations across countries and in rural and urban settings, related primarily to community engagement approaches, informed consent, measuring of household goods as a proxy for socioeconomic status and to request information on HIV status.

The data generated in Phase 1 of the AWI-Gen study could be used for improved modelling of NCD risk in Africa in different regions and in a global health context and this may contribute to the development of policies more appropriate to SSA. Given the paucity of research into the genomic risk for non-communicable diseases in African populations, the AWI-Gen study provides a unique resource to explore genetic associations with cardiometabolic risk factors and endpoints in SSA. In combination with genomic data, and taking into consideration the high genetic diversity of African populations, we expect to detect unique gene-gene and gene-environment interactions that could provide clues to the rapid rise of non-communicable diseases, especially cardiometabolic diseases, on the African continent.

## Paper context

This paper describes in detail the methods used to collect data and biospecimens for Phase 1 of the AWI-Gen study, which aims to examine genetic and environmental factors related to cardiometabolic disease (CMD) in Africans. Understanding the unique risk factor profile and long-term health consequences of rapidly changing environmental and demographic conditions, in the context of African genome variation, will inform public health interventions to mitigate the chronic burden of disease, especially for CMD.
