# Supplementary material for: Genomic and environmental risk factors for cardiometabolic diseases in Africa: methods used for Phase 1 of the AWI-Gen population cross-sectional study
Source: Glob Health Action. 2018 Sep 27;11(Suppl 2):1507133. doi: 10.1080/16549716.2018.1507133 (PMC6161608; doi:10.1080/16549716.2018.1507133)
Supplement: Supplemental Material [file ZGHA_A_1507133_SM7184.zip › Supplemenratry Material Part 1 - Info sheet revised .docx]

**Trait Association Study Component**

**INFORMATION SHEET**

**Consenting around the Wits-INDEPTH Partnership project funded through the Human Heredity and Health in Africa Initiative**

**Collection of blood samples for DNA extraction, storage and genomic studies related to studies to traits and disease risk and disease, and urine collection for metabolic studies**

Dear Potential Participant,

We are a group of scientists from the XXX CentreXXX who are working in partnership with other scientists across four countries, Ghana, Burkina Faso, Kenya and South Africa, to understand the risk for developing obesity, diabetes, high blood pressure and other diseases. The main aim of our study is to understand how inherited differences (traits that we get from our parents) influence our health. In order to clearly understand this we first need to understand the differences between people in specific regions of Africa. We call this population structure. Populations are different because of their history (for example, migration and mixing between populations) but also because of their environment (if an inherited change gives the person a health advantage, then people with that change will be more likely to survive and pass the change on to their children).

We would like to invite you to take part in our study. In order to take part, you need to agree to provide a sample of blood, and a urine sample and to have various measurements done on your body and to complete a list of questions related to your health and your life and your environment. You also need to give us permission to use the genetic material (DNA) we get out of your blood for the specific studies to understand population history, your exposure to your environment, your health, your body shape and your risk for developing disease. To do this we break open the cells in the blood to release the DNA (this stands for deoxyribonucleic acid). The DNA contains information to program the development of your body and when something goes wrong with the DNA, this can lead to specific diseases.

Once we have collected the blood sample (27 ml in 5 tubes - the equivalent of less than two tablespoons) we will give it a code and this code will be used to identify your sample during the study. The information that links your name to your code will be safely stored and will not be used in the study.

Taking a blood sample from a vein in your arm may cause a little discomfort and a little pain. This procedure will be done by a qualified nurse, phlebotomist or doctor.

Once the DNA has been taken out of your blood it will be stored in a safe place at the local Research Institution and at the Sydney Brenner Institute for Molecular Bioscience at the University of the Witwatersrand. A small part of the DNA will also be stored in an H3Africa Biobank. There will be strict control about who may use the samples.

From the studies we will be able to reconstruct some of the past history of the group of people who have donated samples to the study. It will tell us if at some time in the past different populations have mixed and also if there was some factor which led to the selection of specific changes in the DNA.

If at some future date we realise there are other studies that we would like to carry out on your DNA samples, such tests would only be performed if permission is given to us by the Human Research Ethics Committee of the University of the Witwatersrand on your behalf. As stated above, no one will know your identity as your sample will be identified by a code. This study may go one for many years (over 5 years) in order to fully understand the connection between DNA changes and the way you look and your possible health.

Your Participation is voluntary, and if you wish not to participate there is no penalty or loss of benefits in any way. You may discontinue participation in this study at any time without penalty or loss of benefits and your DNA sample will be destroyed and your data removed from the database.

Measurements

Your blood pressure, weight, height, waist and hip circumference will be measured. This will take about 10 minutes.

Ultra sound Scans

An ultra sound scan to measure the internal fat in your body will be done. An ultrasound scan uses high frequency sound waves to take pictures of the body.  This is not an invasive procedure, will cause you no pain and is perfectly safe. The actual procedure should not take more than 10 minutes

cIMT(Carotid Intima Media Thickness)

This measurement will be done by a trained technician. It is almost the same as the ultrasound procedure above. You will lie on a flat bed and the technician will gently pass a small hand held instrument up and down your neck. This small instrument will measure if the arteries leading to your brain are healthy. This is not an invasive procedure and the actual procedure should not take more than 20 minutes.

Questionnaires

We will ask you to complete one questionnaire. We will fill in the answers for you. You can skip any question that makes you uncomfortable. Answering these questionnaires should take approximately twenty minutes.

HIV rapid test

The rapid HIV test is a **completely voluntary**, antibody test which will enable you to know your HIV status; positive or negative.  If you decide to have this test, you will be given pre-test counseling by a fully trained registered counsellor and asked to fill in a separate consent form. This test is always strictly confidential and can only happen if you agree. There is no way in which anyone can link your HIV status to you name as all results in this study are coded with a number. No-one including your doctor, family, or work colleagues will be told about this test without your permission. The advantage of a rapid test is that you do not have to return to get your test result. Results will be available when your check out, after all the other tests, measurements and questionnaires are completed.

You will have your finger pricked with a sterile needle and the drop of blood will be tested on specific HIV testing kits to check for HIV antibodies. Test results will be given to you in private when you check out today by a registered trained counsellor. If the report states negative it means that there are no antibodies to HIV. The window period will be explained. If the report states positive, it means that you are HIV positive and that there are antibodies to HIV. You will be given a letter to refer you to a clinic specialising in HIV treatment and you will be given a second test at the clinic to confirm this result.  Sometimes we cannot clearly tell if the results are negative or positive. We will then refer you to a clinic specialising in HIV treatment and you will be given a second test at the clinic to confirm this result.

Urine sample

You will be requested to donate a small urine sample (20ml) as instructed by the field worker in a clean toilet facility. This will be used to test if your kidneys are working well.

Phenotype and DNA sample data management

The scientists who do the research will generate information (data) which will be placed in databases. Some of the information in the databases will be shared with other scientists, possibly around the world. These scientists will not have access to your name and therefore the information will not be linked back to you. It is possible that we may send small amounts of the DNA out of the country where tests will be done that we cannot easily do in South Africa, but that would give us valuable information for our studies. All findings from this study will be reported for the group and so no other persons will know the results for the single individual.

Benefits

The discoveries that come from the studies on your DNA will not be of direct benefit to you and will not be communicated back to you. The discoveries may lead to information that will help us in the future to diagnose disease, understand who is most likely to get ill and how different people behave when they are given medication.  The DNA belongs to you and we are the keepers of the DNA. You may withdraw your sample at any time.

Risks

Since the DNA of every person is different, it is possible that if someone tested your blood and compared it to the data in the databases, they could conclude that the two samples come from the same person.

Should you require further information or wish to withdraw from the study, you may contact us at:

Project coordinator: Ethics Approval Number:

| 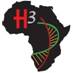 | **AWI-Gen H3Africa**  **Genomic and Environmental Risk Factors for Cardiometabolic Disease in Africans** |
| --- | --- |

**INFORMED CONSENT – Trait Association Study**

**Collection of blood samples for DNA extraction, storage and genomic studies, and urine collection for metabolic studies**

| Name: |  |  |  |  |  |  |  |  |  |  |  |  |  |  |  |  |  |  |  |  |  |  |  |  |  |  |  |  |  |  |
| --- | --- | --- | --- | --- | --- | --- | --- | --- | --- | --- | --- | --- | --- | --- | --- | --- | --- | --- | --- | --- | --- | --- | --- | --- | --- | --- | --- | --- | --- | --- |

| Date of birth: | d | d | / | m | m | / | y | y | y | y |  | Place of birth: |  |
| --- | --- | --- | --- | --- | --- | --- | --- | --- | --- | --- | --- | --- | --- |

The information around the blood sample taken from me and the DNA that will be extracted from the blood is clear and the purpose of consent is for me to inform the study what they can or cannot do with these samples.

| I understand that all procedure/tests on the stored blood and DNA samples will be approved by the Human Research Ethics Committee of the University of the Witwatersrand. | Yes | No |
| --- | --- | --- |
| I am in agreement that my DNA may be stored and used for the purposes described above. | Yes | No |
| I am in agreement that the data generated from my DNA may be made available as stated above. | Yes | No |
| I am in agreement that the information I have supplied in the list of questions and the information from the tests and measurements taken from me may be used as stated above. | Yes | No |
| I agree that a small bit of my DNA may be sent out of the country if the research cannot easily be done in South Africa. | Yes | No |
| I agree that an portion of my DNA may be stored in a repository (laboratory) according to the stipulation of the H3Africa initiative and that some data may be stored in a database as stipulated and that these may be shared according to the processes and procedures of the H3Africa initiative by using my study code or another code that de-identifies my sample and data. | Yes | No |
| I understand that every time a new study is done on my DNA, permission will be obtained from the ethics committee for the study to make sure that it is used only for the purposes stated above. | Yes | No |
| I understand that I will not benefit directly from the research done on my DNA. | Yes | No |
| I understand that I may withdraw from the study at any time. | Yes | No |

| **RESEARCH ASSISTANT** |  |  |  |  |  |  |  |  |  |  |  |  |  | |  | |  | |  | |  | |  |  | |  | |  | |  | |  | |  | |  | |  |  | |  | |  | |  | |  | |
| --- | --- | --- | --- | --- | --- | --- | --- | --- | --- | --- | --- | --- | --- | --- | --- | --- | --- | --- | --- | --- | --- | --- | --- | --- | --- | --- | --- | --- | --- | --- | --- | --- | --- | --- | --- | --- | --- | --- | --- | --- | --- | --- | --- | --- | --- | --- | --- | --- |
|  | Printed Name | | | | | | | | | | | | | | | | | | | | | | | | | | | | | | | | | | | | | | | | | | | | | | |  |
|  |  | | | | | | | | | | | | |  | | d | | d | | / | | m | | | m | | / | | y | | y | | y | | y | | | h | | h | | : | | m | | m | |  |
|  | Signature/Mark or Thumbprint | | | | | | | | | | | | |  |  | Date | | | | | | | | | | | | | | | | | | | | | Time | | | | | | | | | | |  |
|  | | | | | | | | | | | | | | | | | | | | | | | | | | | | | | | | | | | | | | | | | | | | | | | |  |
| **PARTICIPANT** |  |  |  |  |  |  |  |  |  |  |  |  |  | |  | |  | |  | |  | |  |  | |  | |  | |  | |  | |  | |  | |  |  | |  | |  | |  | |  | |
|  | Printed Name | | | | | | | | | | | | | | | | | | | | | | | | | | | | | | | | | | | | | | | | | | | | | | |  |
|  |  | | | | | | | | | | | | |  | | d | | d | | / | | m | | | m | | / | | y | | y | | y | | y | | | h | | h | | : | | m | | m | |  |
|  | Signature/Mark or Thumbprint | | | | | | | | | | | | |  |  | Date | | | | | | | | | | | | | | | | | | | | | Time | | | | | | | | | | |  |
|  | | | | | | | | | | | | | | | | | | | | | | | | | | | | | | | | | | | | | | | | | | | | | | | |  |
| **WITNESS** (if applicable) |  |  |  |  |  |  |  |  |  |  |  |  |  | |  | |  | |  | |  | |  |  | |  | |  | |  | |  | |  | |  | |  |  | |  | |  | |  | |  | |
|  | Printed Name | | | | | | | | | | | | | | | | | | | | | | | | | | | | | | | | | | | | | | | | | | | | | | |  |
|  |  | | | | | | | | | | | | |  | | d | | d | | / | | m | | | m | | / | | y | | y | | y | | y | | | h | | h | | : | | m | | m | |  |
|  | Signature/Mark or Thumbprint | | | | | | | | | | | | |  |  | Date | | | | | | | | | | | | | | | | | | | | | Time | | | | | | | | | | |  |

Study ID: ………………………
