# Supplementary material for: Genomic and environmental risk factors for cardiometabolic diseases in Africa: methods used for Phase 1 of the AWI-Gen population cross-sectional study
Source: Glob Health Action. 2018 Sep 27;11(Suppl 2):1507133. doi: 10.1080/16549716.2018.1507133 (PMC6161608; doi:10.1080/16549716.2018.1507133)
Supplement: Supplemental Material [file ZGHA_A_1507133_SM7184.zip › Supplementary Material Part 2 - AWI-Gen questionnaire.pdf]

AWI-Gen H3Africa

Genomic and Environmental Risk Factors for Cardiometabolic Disease in Africans

Dikgale

**AWI-Gen Study Number: |__|__|__|__||__|**	**Unique Site Identifier : |__|__|__|__||__|__|__|**

**BARCODE STICKER**
Please stick the barcode sticker here

**1. GENERAL INFORMATION**		
**1.1**	Data collection date	|d|d| |m|m|m| |y|y|y|y|
**1.2**	Interviewer code	____________________
**1.3**	Start time	|h|h|:|m|m| ***[Based on a 24 hour clock eg. 15:30]***

**2. DEMOGRAPHIC INFORMATION**		
**2.1**	Date of birth known? ***[If no, skip to Q.2.3]***	☐ Yes ☐ No
**2.2**	Date of Birth ***[eg. 27 SEP 1957]***	|d|d| |m|m|m| |y|y|y|y|
**2.3**	Approximate year of birth	**|1|5| |J|U|N|** |y|y|y|y|
**2.4**	Gender	☐Female ☐Male ***[please tick the appropriate box]***
**2.5**	Country	South Africa
**2.6**	Home language ***[please tick the appropriate box]***	☐ Afrikaans ☐ English ☐ isiNdebele ☐ isiXhosa<br>☐ isiZulu ☐ Sesotho ☐ Sepedi<br>☐ Setswana ☐ siSwati ☐ Tshivenda<br>☐ Xitsonga ☐ Shona ☐ Unknown<br>☐ Other (specify)____________________
**2.7**	Ethnic/tribal affiliation ***[please tick the appropriate box]***	☐ Zulu ☐ Xhosa ☐ Ndebele ☐ Sotho<br>☐Venda ☐ Tsonga ☐ Tswana ☐ BaPedi<br>☐ Shona ☐ Unknown ☐ Swati<br>☐ Other (specify)____________________

2.8. Family Ethnicity ***This question relates to the ethnicity and home languages of your parents and grandparents.***

Please tick the applicable columns

	Father		Paternal Grandfather		Paternal Grandmother		Mother		Maternal Grandfather		Maternal Grandmother	
Ethnicity/Language	E	L	E	L	E	L	E	L	E	L	E	L
Afrikaans												
English												
Ndebele/isiNdebele												
Sotho/Sesotho												
BaPedi/Sepedi												
Swati/siSwati												
Tsonga/Xitsonga												
Tswana/Setswana												
Venda/Tshivenda												
Xhosa/isiXhosa												
Shona/Shona												
Zulu/isiZulu												
Unknown												
Other												

If other, please specify

Father __________________ *Ethnicity* *Language* **PG\father** __________________ *Ethnicity* *Language* **PG\mother** __________________ *Ethnicity* *Language*

Mother __________________ *Ethnicity* *Language* **MG\father** __________________ *Ethnicity* *Language* **MG\mother** __________________ *Ethnicity* *Language*

**3. FAMILY COMPOSITION**		
**3.1**	Do you have any siblings* with which you share at least one parent? ****half siblings and those that have passed away are included***	☐ Yes ☐ No ***[If no, skip to Q.3.4]***
**3.2**	How many brothers do you have?	|__|__|
**3.3**	How many sisters do you have?	|__|__|

**3.4**	Do you have any biological children?	☐ Yes ☐ No *[If no, skip to the next section- Phenotypic Data Collection]*
**3.5**	How many biological sons do you have?	|__|__|
**3.6**	How many biological daughters do you have?	|__|__|

PHENOTYPIC COLLECTION DATA

**4. PREGNANCY** **[If male, please skip to the next section - Marital Status]**		
**4.1**	Are you pregnant?	☐ Yes ☐ No
**4.2**	How many pregnancies have you had? *[If none, please skip to Q.4.4]*	|__|__|
**4.3**	How many live births have you had?	|__|__|
**4.4**	Do you have regular (28-35 day) periods?	☐ Yes ☐ No
**4.5**	Date of last period	|_m_|_m_| |_y_|_y_|_y_|_y_|

**5. MARITAL STATUS**		
**5.1**	Marital Status<br>*[please tick the appropriate box]*	☐ Married<br>☐ Living together<br>☐ Never married or co-habited<br>☐ Divorced with a living partner<br>☐ Separated with a living partner<br>☐ Partner deceased

**6. EDUCATION**		
**6.1**	Highest level of Education<br>*[please tick the appropriate box]*<br>*[If "no formal education" please skip to Q.7]*	☐ No formal education<br>☐ Primary<br>☐ Secondary<br>☐ Tertiary*<br>***tertiary education includes qualifications such as certificates, diplomas or degrees***

6.2	Total number of successfully completed years at highest level of education	**|__|__| years**

7. EMPLOYMENT

7.1	Employment	☐ Self employed<br>☐ Formal full-time employment by someone else<br>☐ Part-time employment by someone else<br>☐ Informal employment (dependent on availability of work)<br>☐ Unemployed

8. HOUSEHOLD ATTRIBUTES

8.1. How many people besides you are in your household? **|__|__| people**

This applies to people who sleep in separate buildings on the property but share their main meal in the same house as you If none, please skip to Q.8.3.

**8.2.**	**Name**	**Sex**	**Age (years)**	**Relationship to you (eg. father, husband, daughter, etc.)**
	1.	☐ Male<br>☐ Female	|__|__|__|	
	2.	☐ Male<br>☐ Female	|__|__|__|	
	3.	☐ Male<br>☐ Female	|__|__|__|	
	4.	☐ Male<br>☐ Female	|__|__|__|	
	5.	☐ Male<br>☐ Female	|__|__|__|	
	6.	☐ Male<br>☐ Female	|__|__|__|	
	7.	☐ Male<br>☐ Female	|__|__|__|	
	8.	☐ Male<br>☐ Female	|__|__|__|	
	9.	☐ Male<br>☐ Female	|__|__|__|	
	10.	☐ Male<br>☐ Female	|__|__|__|	
	11.	☐ Male<br>☐ Female	|__|__|__|	

8.2.	Name	Sex	Age (years)	Relationship to you (eg. father, husband, daughter, etc.)
	12.	☐ Male<br>☐ Female	|__|__|__|	

8.3	How many rooms are there in the house and outside structures used by household members?	**|__|__| rooms**
8.4	How many rooms are used for sleeping in?	**|__|__| rooms**

**8.5**	**Which of the following items in working order, do you have in your household at the present time?**	
**1**	Electricity	☐ Yes ☐ No ☐ Don't know
**2**	Solar energy	☐ Yes ☐ No ☐ Don't know
**3**	Power generator	☐ Yes ☐ No ☐ Don't know
**4**	Alternative power source	☐ Yes ☐ No ☐ Don't know
**5**	Television	☐ Yes ☐ No ☐ Don't know
**6**	Radio	☐ Yes ☐ No ☐ Don't know
**7**	Motor vehicle	☐ Yes ☐ No ☐ Don't know
**8**	Motorcycle	☐ Yes ☐ No ☐ Don't know
**9**	Bicycle	☐ Yes ☐ No ☐ Don't know
**10**	Refrigerator	☐ Yes ☐ No ☐ Don't know
**11**	Washing machine	☐ Yes ☐ No ☐ Don't know
**12**	Sewing machine	☐ Yes ☐ No ☐ Don't know
**13**	Telephone	☐ Yes ☐ No ☐ Don't know
**14**	Mobile phone	☐ Yes ☐ No ☐ Don't know
**15**	Microwave	☐ Yes ☐ No ☐ Don't know
**16**	DVD player	☐ Yes ☐ No ☐ Don't know
**17**	Satellite TV or DSTV	☐ Yes ☐ No ☐ Don't know
**18**	Computer or laptop	☐ Yes ☐ No ☐ Don't know
**19**	Internet by computer	☐ Yes ☐ No ☐ Don't know
**20**	Internet by mobile phone	☐ Yes ☐ No ☐ Don't know
**21**	Electric or gas stove	☐ Yes ☐ No ☐ Don't know
**22**	Toilet facilities	☐ Yes ☐ No ☐ Don't know
**23**	Cattle	☐ Yes ☐ No ☐ Don't know
**24**	Other livestock *	☐ Yes ☐ No ☐ Don't know
**25**	Poultry **	☐ Yes ☐ No ☐ Don't know

8.5	**Which of the following items in working order, do you have in your household at the present time?**	
**26**	Tractor	☐ Yes ☐ No ☐ Don't know
**27**	Plough	☐ Yes ☐ No ☐ Don't know

*** other livestock includes donkeys, goats, sheep and pigs**

**** poultry includes ducks, chickens, geese and other fowl**

9. SUBSTANCE USE

**9.1. Tobacco use**		
**9.1.1**	Have you ever smoked any tobacco products such as cigarettes, cigars or pipes? ***[If no, skip to Q.9.1.8]***	☐ Yes ☐ No
**9.1.2**	Do you *currently smoke any tobacco products, such as cigarettes, cigars or pipes? ****We are asking if they smoke when they have the opportunity to do so.*** ***[If no, skip to Q.9.1.6]*** ***[If yes, please do not answer Q.9.1.7]***	☐ Yes ☐ No
**9.1.3**	What do you smoke? ***[please tick the appropriate boxes]***	☐ Cigarettes ☐ Pipe<br>☐ Hand rolled ☐ Cigars<br>☐ E-cigarettes
**9.1.4**	How often do you smoke tobacco products?	☐ Daily<br>☐ 5-6 days per week<br>☐ 1-4 days per week<br>☐ 1-3 days per month<br>☐ Less than once per month
**9.1.5**	On the days that you smoke, how many tobacco products do you smoke?	|__|__|__|
**9.1.6**	How old were you when you first started smoking?	|__|__| years old
**9.1.7**	When did you stop smoking completely?	|_y_|_y_|_y_|_y_|
**9.1.8**	Have you ever used any smokeless tobacco such as snuff, snus, betel with tobacco or chewing tobacco? ***[If no, skip to Q.9.2]***	☐ Yes ☐ No
**9.1.9**	Do you use snuff? ***[If no, skip to Q.9.1.13]***	☐ Yes ☐ No
**9.1.10**	How do you take snuff?	☐ Through your nose<br>☐ Through your mouth/on your lip

**9.1.11**	How often do you use snuff?	☐ Daily<br>☐ 5-6 days per week<br>☐ 1-4 days per week<br>☐ 1-3 days per month<br>☐ Less than once per month
**9.1.12**	On the days that you use snuff, how many times a day do you use it?	☐ Once a day<br>☐ Twice a day<br>☐ Three times a day<br>☐ More than three times a day
**9.1.13**	Do you use chewing tobacco? ***[If no, skip to Q.9.2]***	☐ Yes ☐ No
**9.1.14**	How often do you use chewing tobacco?	☐ Daily<br>☐ 5-6 days per week<br>☐ 1-4 days per week<br>☐ 1-3 days per month<br>☐ Less than once per month
**9.1.15**	On the days that you use chewing tobacco, how many times a day do you use it?	☐ Once a day<br>☐ Twice a day<br>☐ Three times a day<br>☐ More than three times a day

**9.2. Alcohol use**		
**9.2.1**	Have you ever consumed an alcoholic drink such as beer, wine, spirits, fermented cider, thothotho, or traditional beer? ***[If "no" or "don't know", skip to Q.9.3]***	☐ Yes<br>☐ No<br>☐ Don't know<br>☐ Refuse to answer
**9.2.2**	Do you currently (in the last 30 days) consume any alcoholic drink such as beer, wine, spirits, fermented cider, thothotho, or traditional beer? ***[If no, skip to Q.9.2.10]***	☐ Yes<br>☐ No<br>☐ Don't know<br>☐ Refuse to answer
**9.2.3**	How often do you have at least one alcoholic drink?	☐ Daily<br>☐ 5-6 days per week<br>☐ 1-4 days per week<br>☐ 1-3 days per month<br>☐ Less than once per month
**9.2.4**	On the days that you drink alcoholic drinks, how many alcoholic drinks do you have? USE CARDS TO SHOW STANDARD DRINKS	|__|__|

**9.2.5**	Have you ever felt that you should cut down on your drinking?	☐ Yes<br>☐ No<br>☐ Don't know<br>☐ Refuse to answer
**9.2.6**	Have people annoyed you by criticising your drinking?	☐ Yes<br>☐ No<br>☐ Don't know<br>☐ Refuse to answer
**9.2.7**	Have you ever felt bad or guilty about your drinking?	☐ Yes<br>☐ No<br>☐ Don't know<br>☐ Refuse to answer
**9.2.8**	Have you ever had an alcoholic drink first thing in the morning to steady your nerves or get rid of a hangover?	☐ Yes<br>☐ No<br>☐ Don't know<br>☐ Refuse to answer
**9.2.9**	In the past year, did you ever take 6 or more alcoholic drinks in a single morning, afternoon, or night? I understand that you may share drinks and that some drinks have different sizes, but please do your best to answer. USE CARDS TO SHOW STANDARD DRINKS.	☐ Yes<br>☐ No<br>☐ Don't know<br>☐ Refuse to answer
**9.2.10**	What type of alcoholic beverage do you, or did you, usually drink?	☐ Beer<br>☐ Wine<br>☐ Spirits<br>☐ Home brew<br>☐ Other (specify) ________

**9.3. Drug use**		
**9.3.1**	Do you, or have you ever taken marijuana, methamphetamines, cocaine or any other drugs (dagga, nyaope, tik, glue, rocks, crack, buttons, mandrax, acid)?	☐ Yes<br>☐ No<br>☐ Don't know<br>☐ Refuse to answer

10. GENERAL HEALTH

10.1. Please indicate whether you have, or have had, any of the following illnesses

[Please tick the appropriate boxes]
[If male, please proceed to question 10.1.3]
[If female, please skip question 10.1.3]

10.1.1	Breast cancer ***[for females only]***	☐ Yes	☐ No	☐ Don't know	☐ Refuse to answer
10.1.2	Cervical cancer ***[for females only]***	☐ Yes	☐ No	☐ Don't know	☐ Refuse to answer
10.1.3	Prostate cancer ***[for males only]***	☐ Yes	☐ No	☐ Don't know	☐ Refuse to answer
10.1.4	Other cancers	☐ Yes	☐ No	☐ Don't know	☐ Refuse to answer
10.1.5	Asthma or reactive air diseases	☐ Yes	☐ No	☐ Don't know	☐ Refuse to answer
10.1.6	Have you ever received treatment prescribed by a doctor to treat any of the above illnesses? ***[If no, skip to Q.10.1.9]***	☐ Yes	☐ No	☐ Don't know	
10.1.7	Are you currently on treatment prescribed by a doctor to treat any of the above illnesses? ***[If no, skip to Q.10.1.9]***	☐ Yes	☐ No	☐ Don't know	
10.1.8	What medication has been prescribed? Please list names if possible.	______________________			
10.1.9	Are you currently taking any herbal or traditional remedy for any of the above illnesses?	☐ Yes	☐ No	☐ Don't know	

10.2. Please indicate if your <u>mother</u> has, or has had, any of the following illnesses

[Please tick the appropriate boxes]

10.2.1	Weight problem/obesity	☐ Yes	☐ No	☐ Don't know	☐ Refuse to answer
10.2.2	High blood pressure	☐ Yes	☐ No	☐ Don't know	☐ Refuse to answer
10.2.3	High cholesterol	☐ Yes	☐ No	☐ Don't know	☐ Refuse to answer
10.2.4	Breast cancer	☐ Yes	☐ No	☐ Don't know	☐ Refuse to answer
10.2.5	Cervical cancer	☐ Yes	☐ No	☐ Don't know	☐ Refuse to answer
10.2.6	Other cancers	☐ Yes	☐ No	☐ Don't know	☐ Refuse to answer
10.2.7	Asthma or reactive air diseases	☐ Yes	☐ No	☐ Don't know	☐ Refuse to answer

10.3. Please indicate if your father has, or has had any of the following illnesses

[Please tick the appropriate boxes]

10.3.1	Weight problem/obesity	☐ Yes	☐ No	☐ Don't know	☐ Refuse to answer
10.3.2	High blood pressure	☐ Yes	☐ No	☐ Don't know	☐ Refuse to answer
10.3.3	High cholesterol	☐ Yes	☐ No	☐ Don't know	☐ Refuse to answer
10.3.4	Prostate cancer	☐ Yes	☐ No	☐ Don't know	☐ Refuse to answer
10.3.5	Other cancers	☐ Yes	☐ No	☐ Don't know	☐ Refuse to answer
10.3.6	Asthma or reactive air diseases	☐ Yes	☐ No	☐ Don't know	☐ Refuse to answer

**10.4. Diet**<br><br>***The following questions ask about what you eat and drink.***		
**10.4.1**	In a typical week, on how many days do you eat fruit?	|__| days
**10.4.2**	How many servings of fruit do you eat on a typical day? USE CARDS TO SHOW STANDARD SERVINGS	|__|__| servings
**10.4.3**	In a typical week, on how many days do you eat vegetables?	|__| days
**10.4.4**	How many servings of vegetables do you eat on a typical day? USE CARDS TO SHOW STANDARD SERVINGS	|__|__| servings
**10.4.5**	How many meals per week do you buy from a *vendor or take-away or restaurant? By meal, I mean breakfast, lunch or dinner.<br>***A vendor is any person or place that sells food**	|__|__| meals
**10.4.6**	In a typical week, on how many days do you eat bread bought from a shop?	|__| days
**10.4.7**	How many slices of bread bought from a shop, do you eat on a typical day? USE CARDS TO SHOW STANDARD SLICES	|__|__| slices
**10.4.8**	How many cans, bottles, or cups of sugary cold drinks (eg. Coke, Pepsi, Fanta, Sprite, etc.), do you drink in a week?	|__|__| drinks
**10.4.9**	How many cans or bottles or cups of fruit juice do you drink in a week? (eg. Liquifruit, Tropicana, Oros, etc.)	|__|__| drinks

**10.4.10**	Has a doctor, nurse, or other healthcare worker ever told you to change your diet (eg.To eat less sugar)?	☐ Yes<br>☐ No<br>☐ Don't know
**10.4.11**	Has a doctor, nurse, or other healthcare worker ever advised you to lose weight?	☐ Yes<br>☐ No<br>☐ Don't know

**10.5. Exposure to Pesticides**		
**10.5.1**	Do you work with insecticides or pesticides?<br>***[If no, please skip to Q.10.5.3.]***	☐ Yes<br>☐ No<br>☐ Don't know
**10.5.2**	How long have you been working with insecticides or pesticides?	|__|__| years
**10.5.3**	Do you live close to a farm or region where insecticides or pesticides are used?<br>***[If no, please skip to Q.11]***	☐ Yes<br>☐ No<br>☐ Don't know
**10.5.4**	Do you know what type of pesticides or insecticides are used, either by you, or within your area?<br>***[If not yes, please skip to Q.11]***	☐ Yes<br>☐ No<br>☐ Don't know
**10.5.5**	Please list them if possible :	

11. INFECTION HISTORY

**11.1. Malaria**				
**1**	Have you ever had Malaria? ***[If no, skip to Q.11.2]***	☐ Yes	☐ No	☐ Don't know
**2**	Have you had malaria fever in the last month?	☐ Yes	☐ No	☐ Not sure
**3**	Have you travelled to an area with a *high incidence of malaria, in the last 2 months?<br>****Some areas with high incidence of malaria include; Angola, Burkina Faso, Cameroon, Cote d'Ivoire, DRC, Ghana, Guinea-Bissau, Liberia, Malawi, Sierra Leone, Togo, Coastal or Western Kenya, Uganda and Zambia***	☐ Yes	☐ No	☐ Not sure

11.2. TB		
11.2.1	Have you ever been told by a doctor, nurse or other healthcare worker that you have TB? ***[If no, skip to Q.11.2.7]***	☐ Yes ☐ No ☐ Don't know
11.2.2	Have you been newly-diagnosed with TB in the last 12 months?	☐ Yes ☐ No ☐ Don't know
11.2.3	When was it diagnosed?	|_y_|_y_|_y_|_y_|
11.2.4	Have you ever received treatment for TB prescribed by a doctor, nurse, or other healthcare worker? ***[If no, skip to Q.11.2.7]***	☐ Yes ☐ No ☐ Don't know
11.2.5	Are you currently receiving treatment for TB prescribed by a doctor, nurse, or other healthcare worker?	☐ Yes ☐ No ☐ Don't know
11.2.6	Have you ever been counselled by a doctor, nurse or other healthcare worker, on how you can avoid passing TB onto others ?	☐ Yes ☐ No ☐ Don't know
11.2.7	Are you currently taking any herbal or traditional remedy for TB?	☐ Yes ☐ No ☐ Don't know

12. CARDIOMETABOLIC RISK FACTORS

12.1. Diabetes		
12.1.1	Has a doctor, nurse, or other healthcare worker ever measured your blood or urine for diabetes (sugar in the blood)?	☐ Yes<br>☐ No<br>☐ Don't know
12.1.2	Have you ever been told by a doctor or healthcare worker, that you have diabetes or high blood sugar (outside of pregnancy)?<br>***[The brackets apply only to females]***<br>***[If "no" or "don't know", skip to Q. 12.1.7]***	☐ Yes<br>☐ No<br>☐ Don't know
12.1.3	Have you been newly-diagnosed with diabetes in the last 12 months?	☐ Yes<br>☐ No
12.1.4	Have you ever received treatment for diabetes prescribed by a doctor, nurse, or other healthcare worker?	☐ Yes<br>☐ No<br>☐ Don't know
12.1.5	Are you currently receiving treatment for diabetes prescribed by a doctor, nurse, or other healthcare worker?	☐ Yes<br>☐ No<br>☐ Don't know

**12.1.6**	Are you doing anything to treat your diabetes (sugar in the blood)? *Note: Insulin injections and pills can't be taken at the same time!*	☐ Insulin injection<br>☐ Pills (that you swallow)<br>☐ Special Diet<br>☐ Weight Loss<br>☐ Other (specify) ___________
**12.1.7**	Are you currently taking any herbal or traditional remedy for diabetes (sugar in the blood)?	☐ Yes<br>☐ No<br>☐ Don't know
**12.1.8**	Do you have a family history of diabetes?<br>***[If "no" or "don't know", skip to Q.12.2]***	☐ Yes<br>☐ No<br>☐ Don't know

12.1.9. Which of your family members have, or have had diabetes (sugar in the blood)?

	Mother	☐ Yes	☐ No	☐ Don't know
	Father	☐ Yes	☐ No	☐ Don't know
	Brother 1	☐ Yes	☐ No	☐ Don't know
	Brother 2	☐ Yes	☐ No	☐ Don't know
	Brother 3	☐ Yes	☐ No	☐ Don't know
	Brother 4	☐ Yes	☐ No	☐ Don't know
	Sister 1	☐ Yes	☐ No	☐ Don't know
	Sister 2	☐ Yes	☐ No	☐ Don't know
	Sister 3	☐ Yes	☐ No	☐ Don't know
	Sister 4	☐ Yes	☐ No	☐ Don't know
	Son 1	☐ Yes	☐ No	☐ Don't know
	Son 2	☐ Yes	☐ No	☐ Don't know
	Son 3	☐ Yes	☐ No	☐ Don't know
	Son4	☐ Yes	☐ No	☐ Don't know
	Daughter 1	☐ Yes	☐ No	☐ Don't know
	Daughter 2	☐ Yes	☐ No	☐ Don't know
	Daughter 3	☐ Yes	☐ No	☐ Don't know
	Daughter 4	☐ Yes	☐ No	☐ Don't know
	Other, specify :	☐ Yes	☐ No	☐ Don't know

**12.2. Stroke*** <br>***Jones, W.J., Willams, L. S. and Meschia, J. F. (2001) Validating the Questionnaire for Verifying Stroke-Free Status (QVSFS) by neurological history and examination. Stroke, 32(10): 22232-6. Please note that the word, "physician" has been replaced with the phrase, "doctor, nurse or other healthcare worker" to accommodate the AWI-Gen study.**		
**12.2.1**	Have you ever been told by a doctor, nurse, or other healthcare worker that you have had a stroke?<br><br>***[If "no" or "don't know", skip to Q. 12.2.3]***	☐ Yes<br>☐ No<br>☐ Don't know
**12.2.2**	When was it first diagnosed?<br><br>***[Please indicate in which year your stroke was diagnosed]***	|_y_|_y_|_y_|_y_|
**12.2.3**	Have you ever been told by a doctor, nurse, or other healthcare worker that you have had a ministroke, or transient ischemic attack (TIA)?	☐ Yes<br>☐ No<br>☐ Don't know
**12.2.4**	Have you ever had sudden painless weakness on one side of your body?	☐ Yes<br>☐ No<br>☐ Don't know
**12.2.5**	Have you ever had sudden numbness or a dead feeling on one side of your body?	☐ Yes<br>☐ No<br>☐ Don't know
**12.2.6**	Have you ever had sudden painless loss of vision in one or both eyes?	☐ Yes<br>☐ No<br>☐ Don't know
**12.2.7**	Have you ever suddenly lost one half of your vision?	☐ Yes<br>☐ No<br>☐ Don't know
**12.2.8**	Have you ever suddenly lost the ability to understand what people are saying?	☐ Yes<br>☐ No<br>☐ Don't know
**12.2.9**	Have you ever suddenly lost the ability to express yourself verbally, or in writing?	☐ Yes<br>☐ No<br>☐ Don't know

**12.3. Hypertension**		
**12.3.1**	Has a doctor, nurse, or other healthcare worker ever measured your blood pressure?	☐ Yes<br>☐ No<br>☐ Don't know

**12.3.2**	Have you ever been told by a doctor, nurse, or other healthcare worker that you have hypertension (high blood pressure)?<br>***[If "no" or "don't know", skip to Q. 12.3.7]***	☐ Yes<br>☐ No<br>☐ Don't know
**12.3.3**	Have you been newly-diagnosed with hypertension in the last 12 months?	☐ Yes<br>☐ No<br>☐ Don't know
**12.3.4**	Have you ever received treatment for hypertension prescribed by a doctor, nurse or other healthcare worker?	☐ Yes<br>☐ No<br>☐ Don't know
**12.3.5**	Are you currently on treatment for hypertension prescribed by a doctor, nurse or other healthcare worker?	☐ Yes<br>☐ No<br>☐ Don't know
**12.3.6**	What medicine are you taking for this? Please list if possible.	______________________________
**12.3.7**	Are you currently taking any herbal or traditional remedy for hypertension?	☐ Yes<br>☐ No<br>☐ Don't know

**12.4. Angina***<br>****Angina is characterised as an intense chest pain or discomfort. This pain may occur in the shoulders, arms, neck, back and jaw as well. It may also feel like indigestion. It is considered to be the main symptom of Coronary Heart Disease (CHD).***		
**12.4.1**	Have you ever been told by a doctor, nurse, or other healthcare worker that you have angina (chest pain due to heart disease)?<br>***[If "no" or "don't know", skip to Q. 12.4.5]***	☐ Yes<br>☐ No<br>☐ Don't know
**12.4.2**	Have you ever received treatment for chest pain due to heart disease prescribed by a doctor, nurse or other healthcare worker?	☐ Yes<br>☐ No<br>☐ Don't know
**12.4.3**	Are you currently taking any medication for angina prescribed by a doctor or other healthcare worker for this?	☐ Yes<br>☐ No<br>☐ Don't know
**12.4.4**	What medicine are you taking for this? Please list if possible.	______________________________
**12.4.5**	Are you currently taking any herbal or traditional remedy for angina?	☐ Yes<br>☐ No<br>☐ Don't know

**12.4.6**	During the last 12 months, have you experienced any pain or discomfort in your chest, or pain going to the left arm or neck, when you **walk uphill or hurry**?	☐ Yes<br>☐ No<br>☐ Don't know
**12.4.7**	During the last 12 months, have you experienced any pain or discomfort in your chest, or pain going to the left arm or neck, when you **walk at an ordinary pace on level ground**?<br><br>***[If no to both 12.4.6 and 12.4.7, please skip to Q. 12.5]***	☐ Yes<br>☐ No<br>☐ Don't know
**12.4.8**	What do you do if you get the pain or discomfort when you are walking?	☐ Stop or slow down<br>☐ Rest for a while then carry on<br>☐ Carry on after taking a pain relief medicine that dissolves in your mouth (a nitro spray or tablet)<br>☐ Carry on walking
**12.4.9**	Is the pain or discomfort relieved if you stand still?	☐ Yes<br>☐ No<br>☐ Don't know

12.4.10. Will you show me where you usually experience the pain or discomfort?

Please circle the numbers in the box in the area of the body shown by the participant

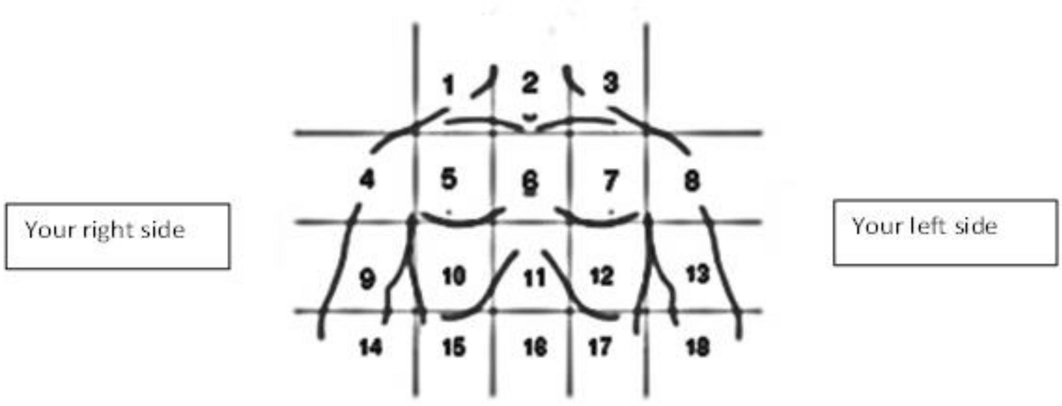

**12.5. Heart Attack**		
**12.5.1**	Have you ever been told by a doctor, nurse, or other healthcare worker that you have had a heart attack?<br>***[If "no" or "don't know", skip to Q. 12.5.3]***	☐ Yes<br>☐ No<br>☐ Don't know

**12.5.2**	Did you ever receive medical treatment for your heart attack?	☐ Yes<br>☐ No<br>☐ Don’t know
**12.5.3**	Are you currently taking any herbal or traditional remedy for your heart attack?	☐ Yes<br>☐ No<br>☐ Don’t know

**12.6. Congestive Heart Failure**		
**12.6.1**	Have you ever been told by a doctor, nurse, or other healthcare worker that you have had heart failure?<br>***[If "no" or "don't know", skip to Q. 12.6.5]***	☐ Yes<br>☐ No<br>☐ Don’t know
**12.6.2**	Have you ever received medical treatment for heart failure prescribed by a doctor, nurse, or other healthcare worker?	☐ Yes<br>☐ No<br>☐ Don’t know
**12.6.3**	Are you currently on treatment for heart failure prescribed by a doctor, nurse, or other healthcare worker?	☐ Yes<br>☐ No<br>☐ Don’t know
**12.6.4**	What medicine are you taking for this? Please list if possible.	________________________
**12.6.5**	Are you currently taking any herbal or traditional remedy for heart failure?	☐ Yes<br>☐ No<br>☐ Don’t know

**12.7. High Cholesterol**		
**12.7.1**	Has a doctor, nurse or other healthcare worker ever measured your cholesterol?	☐ Yes<br>☐ No<br>☐ Don’t know
**12.7.2**	Have you ever been told by your doctor or other healthcare worker told you that you have high cholesterol?<br>***[If "no" or "don't know", skip to Q. 12.7.5]***	☐ Yes<br>☐ No<br>☐ Don’t know
**12.7.3**	Have you ever been treated for high cholesterol by a doctor, nurse, or other healthcare worker?	☐ Yes<br>☐ No<br>☐ Don’t know

**12.7.4**	Are you currently using any of the following to treat your high cholesterol, as prescribed by a doctor, nurse, or other healthcare worker?<br><br>***[Please tick the appropriate boxes, more than one may be selected]***	☐ Special diet<br>☐ Weight loss<br>☐ Medicine<br>☐ Other
**12.7.5**	Are you currently taking any herbal or traditional remedy for high cholesterol?	☐ Yes<br>☐ No<br>☐ Don't know

**13. THYROID DISEASE*** <br>***This includes underactive thyroid, over active thyroid, thyroid nodule enlargement and thyroid cancer***		
**13.1**	Has a doctor ever told you that you have thyroid disease?<br><br>***[If no, skip to Q. 13.5]***	☐ Yes<br>☐ No<br>☐ Don't know
**13.2**	Do you know what type of thyroid disease you were diagnosed with?<br>If yes, please specify	☐ Yes<br>☐ No<br>Specify ________________
**13.3**	Have you ever been treated for it?<br><br>***[If no, skip to Q. 13.5]***	☐ Yes<br>☐ No<br>☐ Don't know
**13.4**	What treatment did you use?	☐ Thyroid hormone<br>☐ Surgery<br>☐ Radioactive iodine<br>☐ Antithyroid drugs
**13.5**	Do either of your parents have, or have they had, thyroid disease? If yes, please specify.<br><br>***[If yes please indicate which parent eg.mother, father or both]***	☐ Yes<br>☐ No<br>Specify ________________

**14. KIDNEY DISEASE**<br><br>***The following questions are aligned with the H3Africa Kidney Disease Study***		
**14.1**	Has a doctor ever told you that you have kidney disease?<br><br>***[If no, skip to Q. 14.3]***	☐ Yes<br>☐ No<br>☐ Don't know

**14.2**	Do you know what type of kidney disease? If yes please specify.	☐ Yes ☐ No<br>Specify ____________
**14.3**	Has a doctor ever told you that your kidneys have low function?	☐ Yes<br>☐ No<br>☐ Don't know
**14.4**	Has anyone in your family either had kidney disease, or died from it?<br><br>***[If no, please skip to Q.15]***	☐ Yes<br>☐ No<br>☐ Don't know
**14.5**	If yes, who?<br><br>***["Other" refers to any other blood relatives eg. Sister, aunt etc]***	☐ Mother<br>☐ Father<br>☐ Other (specify) ____________
**14.6**	Do you know what kind of kidney disease he or she had? If yes, please specify.	☐ Yes ☐ No<br>Specify ____________

15. PHYSICAL ACTIVITY

The following questions are about the time you spend doing different types of physical activities. This includes activities you do at home, at work, travelling from place to place and during your spare time. Work can be paid or unpaid. You are requested to answer the questions even if you don't consider yourself an active person.

**15.1.1**	How many days do you work per week?	|__| days
**15.1.2**	Do you work over the weekend?	☐ Yes ☐ No

15.2. Occupation-related Physical Activity (paid or unpaid work)

The following questions have been aligned with the validated GPAQ for Physical Activity. Respondents should consider their activity during a usual week. Think first about the time you spend doing work. Think of work as the things that you have to do such as paid or unpaid work, study/training, household chores, harvesting food/crops, fishing or hunting for food, seeking employment. In the following questions 'vigorous-intensity activities' are activities that require hard physical effort and cause large increases in breathing or heart rate, 'moderate-intensity activities' are activities that require moderate physical effort and cause small increases in breathing or heart rate. PLEASE USE THE SHOWCARDS FOR PHYSICAL ACTIVITY TO ANSWER THESE QUESTIONS

**15.2.1**	Does your work involve mostly sitting or standing still, or walking for very short periods (less than 10 minutes)?<br><br>***[If yes, skip to Q. 15.2.8]***	☐ Yes ☐ No

**15.2.2**	Does your work involve **vigorous** activities (heavy lifting, digging, manual labour or construction) for at least 10 minutes at a time?<br><br>***[If no, skip to Q.15.2.5]***	☐ Yes ☐ No
**15.2.3**	In a usual week, how many days are spent doing **vigorous** activities as part of your work?	|__| days
**15.2.4**	On a usual day of **vigorous** work, how many hours are spent doing these activities?	|__|__| hrs |__|__| mins
**15.2.5**	Does your work involve **moderate-intensity** activities (brisk walking or carrying light loads) for at least 10 minutes at a time?<br><br>***[If no, skip to Q.15.3]***	☐ Yes ☐ No
**15.2.6**	In a usual week, how many days are spent doing **moderate-intensity** activities at work?	|__| days
**15.2.7**	On a usual work day, how many hours are spent doing **moderate-intensity** activities?	|__|__| hrs |__|__| mins
**15.2.8**	How long is your usual work day?<br><br>***[Please indicate how many hours you work per day on average]***	|__|__| hrs |__|__| mins

15.3. Travel-related physical activity

The following questions have been aligned with the validated GPAQ for Physical Activity. Respondents should consider their activity during a usual week. These questions exclude the physical activities at work that you have already mentioned. These questions are about the usual way you travel to and from places. For example to work, for shopping, to market, to place of worship. PLEASE USE THE SHOWCARDS FOR PHYSICAL ACTIVITY TO ANSWER THESE QUESTIONS

**15.3.1**	Do you walk or use a bicycle (for at least 10 minutes at a time) to get to and from places?<br>***[If no, please skip to Q.15.4.]***	☐ Yes ☐ No
**15.3.2**	In a usual week, how many days do you walk or cycle for at least 10 minutes to get to and from places?	|__| days
**15.3.3**	On a usual day, how many hours do you spend walking or cycling for travel?	|__|__|hrs |__|__| mins

15.4. Non-work related and leisure time Physical Activity

The following questions have been aligned with the validated GPAQ for Physical Activity. Respondents should consider their activity during a usual week. The next questions, exclude the work and transport activities that you have already mentioned, they are about sports, fitness and recreational activities (leisure). PLEASE USE THE SHOWCARDS FOR PHYSICAL ACTIVITY TO ANSWER THESE QUESTIONS

**15.4.1**	In your spare time, do you engage in any **vigorous** or **moderate-intensity** physical activities lasting more than 10 minutes at a time?<br><br>***[If no, skip to Q. 15.5]***	☐ Yes ☐ No

**15.4.2**	In your spare time do you do any **vigorous** activities like running, strenuous sport or exercise for at least 10 minutes at a time?<br><br>***[If no, skip to Q.15.4.5]***	☐ Yes ☐ No
**15.4.3**	In a usual week, how many days do you engage in **vigorous** activities as part of your leisure time?	|__| days
**15.4.4**	In a normal day, how many leisure hours are spent doing **vigorous** activities?	|__|__| hrs |__|__| mins
**15.4.5**	In your spare time, do you engage in any **moderately intense** physical activities like walking or swimming for at least 10 minutes at a time?<br><br>***[If no, skip to Q.15.5]***	☐ Yes ☐ No
**15.4.6**	In a normal week, how many days are spent engaging in **moderately intense** physical activity as part of your leisure time?	|__| days
**15.4.7**	How many leisure hours are spent doing **moderate-intensity** activities in a normal day?	|__|__| hrs |__|__| mins

**15.5. Sitting/Resting Activity**<br><br>***The first question has been aligned with the validated GPAQ for Physical Activity. Respondents should consider their activity during a usual week. A usual day is defined as a weekday.***		
**15.5.1**	Over the past 7 days, how many hours did you spend sitting or reclining on a usual day (excluding sleep)? This may include time sitting at a desk, visiting friends, reading, or sitting down to watch television during working hours and leisure or spare time.	|__|__| hrs |__|__|mins
The following questions provide a better understanding of sedentary behaviour.		
**15.5.2**	How many hours per day do you spend **sitting**, while you are at work?	|__|__| hrs |__|__|mins
**15.5.3**	How many hours do you spend sitting **watching TV** per day, during the **week**?	|__|__| hrs |__|__|mins
**15.5.4**	How many hours per day, do you spend **watching TV** during the **weekend**?	|__|__| hrs |__|__|mins
**15.5.5**	How many hours per day, are spent sitting while using a **computer** outside of your normal working hours during the **week**?	|__|__| hrs |__|__|mins
**15.5.6**	How many hours per day are spent using a **computer** during the **weekend**?	|__|__| hrs |__|__|mins
**15.5.7**	How many hours per day do you spend sitting (eg. in car, bus, train) while **travelling** from place to place during the **week**?	|__|__| hrs |__|__|mins
**15.5.8**	How many hours per day are spent sitting while **travelling** from place to place (eg. in car, bus, train) during the **weekend**?	|__|__| hrs |__|__|mins

**15.5.9**	How many hours per day do you spend sitting while **socialising** during the **week**?	|__|__| hrs |__|__|mins
**15.5.10**	How many hours per day do you spend sitting while **socialising** over the **weekend**?	|__|__| hrs |__|__|mins
**15.5.11**	How many hours per day do you spend sitting while **relaxing** during the **week**?	|__|__| hrs |__|__|mins
**15.5.12**	How many hours per day do you spend sitting while **relaxing** during the **weekend**?	|__|__| hrs |__|__|mins
**15.5.13**	How many hours per day are spent sitting while at church during the **week**?	|__|__| hrs |__|__|mins
**15.5.14**	How many hours per day are spent sitting while at church during the **weekend** ?	|__|__| hrs |__|__|mins

16. SLEEP

The following questions relate to how much time is spent asleep per day

[Based on a 24 hour clock eg.15:30]

**16.1**	What time do you go to sleep during the week?	|h|h|:|m|m|
**16.2**	What time do you wake up during the week?	|h|h|:|m|m|
**16.3**	What time do you go to sleep during the weekend?	|h|h|:|m|m|
**16.4**	What time do you wake up during the weekend?	|h|h|:|m|m|

**17.**	Time at completion of questionnnaire	|h|h|:|m|m| ***[Based on a 24 hour clock eg.15:30]***

Please place barcode sticker here	**Unique Site Identifier : |__|__|__|__|__||__|__|__|**

SAMPLE COLLECTION DATA

**1. ANTHROPOMETRIC MEASUREMENTS**		
**1.1**	Standing height	|__|__|__|__|__| mm
**1.2**	Weight	|__|__|__|.|__| kg
**1.3**	Waist circumference	|__|__|__|__|__| mm
**1.4**	Hip circumference	|__|__|__|__|__| mm

**2. BLOOD PRESSURE**		
**2.1**	Systolic 1	|__|__|__| mmHg
**2.2**	Systolic 2	|__|__|__| mmHg
**2.3**	Systolic 3	|__|__|__| mmHg
**2.4**	Diastolic 1	|__|__|__| mmHg
**2.5**	Diastolic 2	|__|__|__| mmHg
**2.6**	Diastolic 3	|__|__|__| mmHg
**2.7**	Time blood pressure taken in the first instance<br>***[Based on a 24 hour clock eg.15:30]***	**|_h_|_h_|:|_m_|_m_|**

**3. PULSE**		
**3.1**	Pulse 1	|__|__|__| beats/min
**3.2**	Pulse 2	|__|__|__| beats/min
**3.3**	Pulse 3	|__|__|__| beats/min
**3.4**	Person performing measurements	______________________________

**3.5**	Notes or comments related to measurements	

4. ULTRASOUND MEASUREMENTS		
**4.1**	Ultrasound	☐ Yes ☐ No
**4.2**	Visceral (medial) fat	|__|__|.|__|__| cm
**4.3**	Subcutaneous (transverse) fat	|__|__|.|__|__| cm
**4.4**	Ultrasound operator name	____________________________
**4.5**	Date ultrasound taken ***[eg. 27 SEP 1957]***	|_d_|_d_| |_m_|_m_|_m_| |_y_|_y_|_y_|_y_|
**4.6**	cIMT	☐ Yes ☐ No
**4.7**	Minimum cIMT on the right	|__|.|__|__| mm
**4.8**	Maximum cIMT on the right	|__|.|__|__| mm
**4.9**	Mean cIMT on the right	|__|.|__|__| mm
**4.10**	Minimum cIMT on the left	|__|.|__|__| mm
**4.11**	Maximum cIMT on the left	|__|.|__|__| mm
**4.12**	Mean cIMT on the left	|__|.|__|__| mm

	**AWI-Gen H3Africa**<br>**Genomic and Environmental Risk Factors for Cardiometabolic Disease in Africans**<br>Dikgale

Please place barcode sticker here	**Unique Site Identifier : |__|__|__|__|__||__|__|__|**

1. BLOOD COLLECTION

**1.1**	Fasting blood? ***[If YES, skip to Q1.3]***	☐ Yes ☐ No
**1.2**	At what time did you last eat? ***[Based on a 24 hour clock eg.15:30]***	|_h_|_h_|:|_m_|_m_|
**1.3**	Fasting confirmed	☐ Yes ☐ No

1.4. ***Please complete the following table***

**4ml or 6ml Tubes**	**Number**		**Reason**	**Notes**
**Red**	**2**	☐ Yes ☐ No	**lipid**	
**Purple**	**2**	☐ Yes ☐ No	**DNA extraction**	
**Grey**	**1**	☐ Yes ☐ No	**glucose**	

**1.5**	Phlebotomist Name	______________________
**1.6**	Date blood taken ***[eg. 27 SEP 1957]***	|_d_|_d_| |_m_|_m_|_m_| |_y_|_y_|_y_|_y_|
**1.7**	Time blood taken	|_h_|_h_|:|_m_|_m_|

2. HIV

**2.1**	Have you ever been tested for HIV?<br>***[If "no" or "don't know", skip to Q 2.5]***	☐ Yes ☐ Don't know<br>☐ No ☐ Refuse to answer
**2.2**	Do you know your status?<br>***[If "no" or "don't know", skip to Q.2.5.]***	☐ Yes ☐ Don't know<br>☐ No ☐ Refuse to answer
**2.3**	Have you ever tested HIV positive?<br>***[If "no" or "don't know", skip to Q.2.5.]***	☐ Yes ☐ Don't know<br>☐ No ☐ Refuse to answer

**2.4**	Do you use medication prescribed by a doctor, nurse or healthcare worker to treat it?	☐ Yes ☐ Don't know<br>☐ No ☐ Refuse to answer
**2.5**	Are you currently taking any herbal or traditional remedy for HIV?	☐ Yes ☐ Don't know<br>☐ No ☐ Refuse to answer
**2.6**	Do you agree to have your blood sample tested for HIV?<br>***[If no, please mark Q. 2.6 as NA]***	☐ Yes ☐ No
**2.6**	Result	☐ Positive ☐ Negative ☐ NA

3. Test Results

Please complete the table below

**Test**	**Result**	**Notes**
**Fasting Plasma Glucose**		
**Fasting Insulin**		
**HbA1c**		
**HDL**		
**LDL**		
**Triglycerides**		
**Total Cholesterol**		

4. Urine Collection

**4.1**	Urine sample taken	☐ Yes ☐ No

Please place barcode sticker here	**Unique Site Identifier : |__|__|__|__||__|__|__|**

CHECKLIST

**1**	Informed consent	☐ Yes ☐ No
**2**	Questionnaire	☐ Yes ☐ No
**3**	Anthropometric measurements	☐ Yes ☐ No
**4**	Blood pressure	☐ Yes ☐ No
**5**	Pulse	☐ Yes ☐ No
**6**	Blood samples	☐ Yes ☐ No
**7**	Ultrasound	☐ Yes ☐ No
**8**	cIMT	☐ Yes ☐ No
**9**	HIV test	☐ Yes ☐ No
**10**	Urine sample	☐ Yes ☐ No
**11**	Quality controller ID	|__|__|__|__|__|__|__|__|__|__|__|__|
**12**	Travel Reimbursement	☐ Yes ☐ No
